# Supplementary material for: Prevalence and associated risk factors of HIV infections in a representative transgender and non-binary population in Flanders and Brussels (Belgium): Protocol for a community-based, cross-sectional study using time-location sampling
Source: PLoS One. 2022 Apr 11;17(4):e0266078. doi: 10.1371/journal.pone.0266078 (PMC9000107; doi:10.1371/journal.pone.0266078)
Supplement: S1 Appendix — (PDF) [file pone.0266078.s002.pdf]

APPENDIX A

First questionnaire

1. Background information

a. What year were you born? (drop down list)

b. In what country were you born? (dropdown list with Belgium at the top)

c. Was one of your biological parents born abroad?
   i. Yes
   ii. No

d. What is the country of birth of your biological mother? (dropdown list countries with option 'unknown')

e. What is the country of birth of your biological father? (dropdown list countries with option 'unknown')

f. What is the postcode of your current primary residence? By primary residence, we mean the place where you stay or live most of the time. This may differ from your official address. (open field with requirement to enter 4 digits)

g. In order to analyse the data from this survey, we need to ask for the gender category with which you identify yourself. That is why we would like to ask you which group most closely matches what you currently call yourself? Select the answer that best fits you at the moment.
   i. (trans) man
   ii. (trans) woman
   iii. non-binary/gender fluid/gender queer
   iv. transvestite/cross-dresser
   v. I don't know

h. In order to correctly interpret the data, we also need to ask for your assigned birth gender. What is the gender assigned to you at birth (registered on the birth certificate)?
   i. male
   ii. female
   iii. other, namely:

i. Which of the following locations or events where transgender and non-binary people come together have you visited? You can select multiple answers.
   i. Talk group(s)

ii. Gatherings (e.g. reception activities, film night, game night, poetry evenings, holebibib)
iii. Nightlife (e.g. LGBT+ cafes or bars, receptions, parties, dance evenings)
iv. Major events (e.g. T-day, Pride, Sparkle...)
v. Locations related to sex work
vi. Other:

j. How often do you visit the selected locations in non-COVID-19 times?
   i. Less than once monthly
   ii. Once per month
   iii. Two to three times a month
   iv. Once per week
   v. Two to three times a week
   vi. Every day

k. Are you a member of social media groups or forums that mainly include non-binary and/or transgender people? Think for example of TG België, Brotherhood, Discord T-Jong, Queer it up...
   i. Yes
   ii. No

l. How many of these different social media groups or forums are you a member of?
   i. 0 to 3
   ii. 3 to 6
   iii. 6 to 9
   iv. more than 9

m. Have you ever been in contact with a care provider (psychologist, endocrinologist, surgeon, speech therapist, other care provider) who specializes in transgender care?
   i. No, never
   ii. Yes, previously but not any longer
   iii. Yes, I am currently in counseling
   iv. I am on a waiting list to start counseling

n. How often are you currently in contact with these transgender care providers?
   i. Less than once a year
   ii. Once per year
   iii. Two to three times a year
   iv. About six times a year
   v. Once per month
   vi. More than once a month

2. Sexual contacts

In this part, we ask about your sexual contacts in the past period. By 'sexual contact', we mean here all kinds of lovemaking involving genital contact, i.e. touching someone else's genitals, whether orally, vaginally or anally.

a. Have you had sexual contact with at least 1 person in the last 12 months?
   i. yes
   ii. no

b. Did you have a new sex partner in the last 4 months?
   i. Yes
   ii. No

c. Have you had multiple sex partners in the last 12 months?
   i. Yes
   ii. No

d. Have you had sexual contact with more than one person at a time (group sex) in the last 12 months?
   i. Yes
   ii. No

e. With whom have you had sexual contact in the last 12 months? (multiple choice and multiple answers possible)
   i. Cisgender man or men
   ii. Cisgender woman or women
   iii. Transgender man or men
   iv. Transgender woman or women
   v. Non-binary person(s)
   vi. I don’t know

f. Have you paid for sexual contact in the last 12 months?
   i. Yes
   ii. No

g. Have you received goods or money in exchange for sexual contact in the last 12 months?
   i. Yes
   ii. No

h. To the best of your knowledge, have you had sexual contact in the last 12 months with a partner who: (multiple choice and multiple answers possible)
   i. paid for sex in the last 12 months
   ii. received goods or money in exchange for sex in the last 12 months
   iii. ever injected drugs
   iv. is infected with HIV

v. is infected with a sexually transmitted disease other than HIV
vi. was born abroad
vii. participated in group sex in the last 12 months
viii. had multiple sex partners in the last 12 months
ix. had anal sex with other partners in the last 12 months
x. none of the above
xi. I don't know

i. In which country or countries was or were your sexual partner(s) born? (dropdown list with countries and option 'I don't know')

j. Have you had any of the following sexual contacts in the last 12 months?
   i. Receptive anal sex (= bottom, receiving partner)
   ii. Insertive anal sex (= top, giving partner)
   iii. None of the above

k. Did you or your sex partner use a condom?
   i. Always
   ii. Mostly
   iii. Sometimes
   iv. Never

3. Sexually transmitted infections and hiv

a. Have you ever been diagnosed with a sexually transmitted disease (STD)? This may include HIV, hepatitis B or C, chlamydia, genital/anal warts, genital/anal herpes, gonorrhoea, syphilis or others.
   i. Yes
   ii. No

b. Which sexually transmitted infection (STD) have you already been diagnosed with? You can select multiple answers.
   i. HIV
   ii. Hepatitis B
   iii. Hepatitis C
   iv. Chlamydia
   v. Genital/anal warts
   vi. Genital/anal herpes
   vii. Gonorrhoea
   viii. Syphilis
   ix. Crab lice
   x. Other:

c. Do you use antiretroviral therapy (ART) to treat HIV?
   i. Yes

ii. No

d. Have you ever been tested for HIV infection?
   i. Yes
   ii. No

e. When was your last hiv test?
   i. 0-3 months ago
   ii. 3-6 months ago
   iii. 6-12 months ago
   iv. 1-5 years ago
   v. More than 5 years ago
   vi. I don’t know

f. How was this most recent HIV test performed?
   i. GP
   ii. Hospital
   iii. Medical Service
   iv. Swab2know
   v. Self-check
   vi. Other: (please specify)

g. Have you ever used PrEP medication for HIV infection prevention?
   i. Yes
   ii. No
   iii. I don't know what PrEP means

4. Other questions

a. Have you ever injected any of the following yourself: drugs, silicone, testosterone, or muscle-enhancing/performance-enhancing drugs (e.g. anabolic steroids)?
   i. Yes
   ii. No

b. Can you indicate which of the following substances you have ever used:
   i. drugs
   ii. silicone
   iii. testosterone injections
   iv. other muscle-strengthening/performance-enhancing drugs (e.g. anabolic steroids)

c. Have you had any needle treatment in the last 4 months: tattoo, piercing, acupuncture, permanent make-up, perforations?
   i. Yes

ii. No

d. In the past 4 months, have you had contact with another person's blood as a result of a puncture, bite, cut or splash accident?
   i. Yes
   ii. No

5. Contact

a. Do you still need to receive a test kit (material for taking a saliva sample)? (Only mark "yes" if you have not yet taken a saliva sample).

b. May we send you a follow-up questionnaire via email, including questions about your experience with sex, body experience, safe sex, and needs with regard to sexual health?
   i. yes
   ii. no

c. Would you like to be kept informed of the results of this survey by email?
   i. yes
   ii. no

Thank you for participating in this study!
