# Supplementary material for: Prevalence and associated risk factors of HIV infections in a representative transgender and non-binary population in Flanders and Brussels (Belgium): Protocol for a community-based, cross-sectional study using time-location sampling
Source: PLoS One. 2022 Apr 11;17(4):e0266078. doi: 10.1371/journal.pone.0266078 (PMC9000107; doi:10.1371/journal.pone.0266078)
Supplement: S2 Appendix — (PDF) [file pone.0266078.s003.pdf]

Appendix B

Follow-up questionnaire

1. Medical background questions

a. Have you undergone an operation, keyhole surgery or endoscopic examination in the past 4 months?
   i. Yes
   ii. No

b. Have you ever had a blood transfusion, tissue or organ transplant?
   i. Yes
   ii. No

c. (only if 1b = yes): In which country was that? (drop-down list)

d. Have you used drugs in the past 12 months?
   i. Yes
   ii. No
   iii. Prefer not to say

e. (only if 1d = yes): Which drugs have you used in the past 12 months? You can select multiple answers.
   i. Cannabis (weed, marijuana)
   ii. Cocaine
   iii. Amphetamines/speed
   iv. Ecstasy/MDMA
   v. LSD/Acids
   vi. Heroin
   vii. Methadone
   viii. Morphine plaster
   ix. Poppers
   x. Other, namely: (text box)

2. Demographic background questions

a. What is the highest level of education you have completed? If you are currently following a course, you may choose that level.
   i. None
   ii. Elementary education (primary school)
   iii. Lower secondary education (first 3 years completed)
   iv. Upper secondary education (6 years completed)

    v. Higher education (bachelor, graduate, candidacy)
    vi. University education (master, licentiate, postgraduate)
    vii. Master after master
    viii. Doctorate

b. Which of these situations currently best describes your current work situation? You can select multiple answers.
    i. At work (or temporary leave status)
    ii. Unemployed/in search of employment
    iii. Long term illness/disabled
    iv. Independent/entrepreneur
    v. Retired (including early and pre-retirement)
    vi. Responsible for everyday shopping and taking care of the household
    vii. Student
    viii. Other, namely: (text box)

c. Who do you always or usually live with? You can select multiple answers.
    i. No one
    ii. Partner(s)
    iii. Child(ren)
    iv. Parent(s)
    v. Other family member(s)
    vi. Friend(s)
    vii. Other, namely: (text box)

d. What is your current relationship status? Mark each appropriate answer.
    i. Single
    ii. I have a partner but I don't live with them
    iii. I have a partner and I live with them or am married
    iv. I am divorced and not in a new relationship
    v. I am divorced and in a new relationship
    vi. I am a widow(er)
    vii. I have multiple partners
    viii. Other, namely: (text box)

e. A household can have several sources of income, and more than one member of the family can contribute. When you think about all the sources of income your household has, how easy or difficult is it for your household to make ends meet?
    i. very easy
    ii. easy
    iii. fairly easy
    iv. with a little effort
    v. difficult
    vi. very difficult

f. We would like to know who you are <u>currently</u> sexually/romantically attracted to. I am attracted to… (Check everything that applies to you).
   i. (Cisgender) men
   ii. (Cisgender) women
   iii. Transgender and/or non-binary persons
   iv. No one
   v. I don't care about gender
   vi. I don´t know
   vii. Other, namely: (text box)

g. How would you <u>currently</u> describe your sexual orientation?
   i. Heterosexual
   ii. Homosexual
   iii. Lesbian
   iv. Bi(+)
   v. Asexual
   vi. Pansexual
   vii. Other, namely: (text box)

h. Are you <u>currently</u> living according to your perceived gender identity?
   i. Always
   ii. Almost always
   iii. Occasionally
   iv. Never

i. (if **2h = never or occasionally**): Do you plan to do this or more in the future?
   i. yes
   ii. no
   iii. not sure yet

j. Have you had your gender registration adjusted on your identity documents/birth certificate?
   i. Yes
   ii. No

k. (if **2y = no**) Is there a reason why you haven't done this (yet)? You can indicate everything that applies to you.
   i. I don't want to.
   ii. I am satisfied with my current gender registration.
   iii. I don't think this is necessary.
   iv. I want to do this in the future.
   v. I don't know if I can do this.
   vi. I find the procedure too difficult or too expensive.
   vii. My application has been declined.
   viii. The gender designation with which I wish to identify myself is (currently) not an option in the law.

ix. Other, namely: (text box)

l. Do you <u>currently</u> use hormones (such as testosterone and oestrogen) to confirm your gender identity?
   i. yes
   ii. no, but I used to
   iii. No, no need for that
   iv. No, but I would like to do this in the future
   v. I don't know

m. (only **if 2l = yes**): Is your <u>current</u> hormone use medically monitored and checked?
   i. yes, by an endocrinologist
   ii. yes, by a general practitioner
   iii. no
   iv. Other, namely: (text box)

n. (only if **2l = ii: no, but used to be**): Was this hormone use medically monitored and checked <u>when you took hormones</u>?
   i. yes, by an endocrinologist
   ii. yes, by a general practitioner
   iii. no
   iv. other, namely: (insert box)

o. Have you <u>ever</u> had your body changed through a gender-affirming surgical procedure? By 'gender confirming surgery' we mean here <u>all</u> procedures that were part of your (medical) transition.
   i. No, no need
   ii. No, but I would like to do this in the future
   iii. No, and I don't know if I want to.
   iv. Yes, one operation
   v. Yes, one to multiple operations

p. (only if **2o = iv and v**): Which of the following gender-affirming surgical procedures have you already had?
   i. facial surgery
   ii. breast removal
   iii. breast augmentation
   iv. vaginoplasty
   v. metoidioplasty
   vi. phalloplasty
   vii. removal of uterus and/or ovaries
   viii. removal of testes
   ix. Other, namely: (text box)

3. Solo sex

a. Have you ever masturbated yourself?
   i. Yes
   ii. No
   iii. Prefer not to say

b. (only if **3a = yes**): How old were you when you first masturbated? (list of numbers/ages + option at the top 'Don't remember').

c. (only if **3a = no**): Can you indicate for what reason you have never masturbated yourself? You can select multiple answers.
   i. No need
   ii. Because I experience discomfort with my genitals
   iii. Due to physical health reasons
   iv. I don't know how to satisfy myself
   v. I don't find masturbating exciting
   vi. No time for this.
   vii. Other, namely: (text box)

d. Has anyone ever masturbated you?
   i. Yes
   ii. No
   iii. Prefer not to say

e. (only if **3d = yes**): How old were you when someone first masturbated you? (list of numbers/ages + option at the top 'Don't remember').

f. (only if **3d = no**): Can you indicate for what reason no one has ever masturbated you? You can select multiple answers.
   i. No need
   ii. Because I experience discomfort with my genitals
   iii. Due to physical health reasons
   iv. Others don't know how to satisfy me
   v. Others have no respect for my body
   vi. I don't find being masturbated arousing
   vii. No time for this.
   viii. Other, namely: (text box)

4. Sexual contacts

a. Have you ever had sexual contact with someone else? By 'sexual contact', we mean here all kinds of lovemaking involving genital contact, i.e. touching someone else's genitals, whether orally, vaginally or anally.
   i. Yes
   ii. No

b. (only if **4a = yes**): How old were you when you <u>first</u> had sexual contact with someone else? (dropdown list with ages/numbers)

c. (only if **4a = yes**): Have you had sexual contact with someone else in the <u>past six months</u>?
   i. Yes
   ii. No

d. (only if **4c = yes**): How often did you have sexual contact with someone else in the <u>past six months</u>?
   i. Once in the last six months
   ii. About once a month
   iii. Few times a month
   iv. About once a week
   v. Several times a week
   vi. On a daily basis

e. (only if **4a = yes**): Did you use your genitals during this sexual contact?
   i. Yes and I enjoy it as well
   ii. Yes, but I don't enjoy it
   iii. People may sometimes touch it
   iv. People may never touch it
   v. Other, namely: (text box)

f. (only if **4a = yes**): Do you <u>currently</u> have (a) steady sexual partner(s)?
   i. Yes
   ii. No

g. How satisfied or dissatisfied are you currently with your sex life <u>over the past six months</u>?
   i. Very satisfied
   ii. Satisfied
   iii. Neutral
   iv. Dissatisfied
   v. Very dissatisfied

h. (only if **4g = v: very dissatisfied**): You answered 'Very dissatisfied' to the previous question. Can you clarify this? (Open answer)

i. How satisfied or dissatisfied are you with the <u>number of times</u> you've had sex in the <u>past six months</u>?
   i. Very satisfied
   ii. Satisfied
   iii. Neutral
   iv. Dissatisfied
   v. Very dissatisfied

j. In the past six months, have you ever been bothered by the following situations?
   i. Don't feel like engaging in sexual activities
   ii. Difficulty getting excited or staying excited
   iii. Not cumming
   iv. Cumming too soon
   v. Pain during sex
   vi. None of the above
   vii. Other, namely: (text box)

k. What is important or less important to you in sexual contact or in (a) sexual relationship(s)?

	**Not at all important**	**Not important**	**Neutral**	**Important**	**Very important**	**Not applicable/I don't know**
That I can talk about sex and my desires	O	O	O	O	O	O
That I am interested in sex on the same level as my partner(s)	O	O	O	O	O	O
That we decide as equals about how, where and when we have sex	O	O	O	O	O	O
That my gender identity is respected	O	O	O	O	O	O
That my body is respected	O	O	O	O	O	O
That we protect ourselves against sexually transmitted infections (STIs)	O	O	O	O	O	O
That we are regularly tested for sexually transmitted diseases	O	O	O	O	O	O
That we protect ourselves with contraceptives if we don't want to get pregnant/have children	O	O	O	O	O	O

l. (only if **4a = yes**): Thinking about the last time you had sexual contact, do you agree or disagree with the following statements?

	**Strongly disagree**	**Disagree**	**Neither disagree nor agree**	**Agree**	**Strongly agree**	**I don't know/I'd rather not say**
I had sex the way I wanted	O	O	O	O	O	O
I had consensual sex	O	O	O	O	O	O
I felt that my body was accepted during sex	O	O	O	O	O	O
I felt that my boundaries were respected during sex	O	O	O	O	O	O
I felt I could suggest and use a condom if I wanted to	O	O	O	O	O	O

I had sex in a safe place where I felt safe	O	O	O	O	O	O

m. (only if 4a = yes): Which of the following sexual acts did you perform the last time you had sex with someone else? You can indicate all the options that apply to you.
   i. Stroking, jerking, fingering
   ii. Oral sex (blowjob, pussy eating, …)
   iii. Dry sex (clothed sex that involves rubbing the genitals against the body or genitals of the other person)
   iv. Vaginal sex: penetration with (neo-)penis
   v. Vaginal sex: device/sex toy penetration
   vi. Anal sex: penetration with (neo-)penis
   vii. Anal sex: penetration with tool/sex toy
   viii. Other, namely: (text box)

n. Do you ever avoid sexual contact with someone else because of an uneasy feeling towards your own body?
   i. Never
   ii. sometimes
   iii. Several times
   iv. almost always
   v. always

o. (only if **4n = ii, iii, iv or v**): Can you indicate which parts of your own body you feel uncomfortable with, so that you sometimes avoid sexual contact? You can select multiple answers.
   i. Head hair
   ii. Facial hair
   iii. Body hair
   iv. Nose
   v. Chin
   vi. Ears
   vii. Mouth/lips
   viii. Voice
   ix. Shoulder(s)
   x. Chest area
   xi. Stomach
   xii. Hips
   xiii. Glutes
   xiv. Calves
   xv. Arms
   xvi. Hands
   xvii. Penis
   xviii. Balls/testicles

xix. Vagina
xx. Clitoris
xxi. Body weight
xxii. Body height
xxiii. Muscles
xxiv. Feet
xxv. Other, namely:

p. (only if 4a= yes): Can you indicate how often you agree with the statements below or how often you think the statements would be correct for you?

	Strongly disagree	Disagree	Neither disagree nor agree	Agree	Strongly agree	Prefer not to say/Not applicable
The first time I have sex with a new partner, I (would) be concerned that my partner will be put off by seeing my body without clothes.	O	O	O	O	O	O
The idea of having sex without any covering on my body makes me anxious.	O	O	O	O	O	O
The worst thing about having sex is being completely naked in front of another person.	O	O	O	O	O	O
During sex it is (would be) hard not to think about how unattractive my body is.	O	O	O	O	O	O
I (could) only feel comfortable enough to have sex when it was dark so that my partner couldn't see my body clearly.	O	O	O	O	O	O

5. Protected sex

a. (only if **4a = yes**): Which of the following forms of protection did you use during your most recent sexual contact with someone else? Multiple answers are possible.
i. None
ii. Birth control pill
iii. The mini pill
iv. The contraceptive pill
v. The plaster (Evra)
vi. The vaginal ring (Nuvaring)
vii. A hormone IUD
viii. Condom for people with a penis
ix. Condom for people with vagina
x. Diaphragm (rubber dome)
xi. Periodic abstinence or other 'rhythm' methods: no sex during fertile period
xii. Coitus interruptus: withdrawing penis before ejaculation
xiii. Other, namely: (text box)

b. (only if **5a= none**): You did not use <u>any</u> contraceptive during your most recent sexual contact, what is the reason for that? You can select multiple answers.
   i. I don't know how to use a contraceptive
   ii. I don't like it.
   iii. I have a steady partner
   iv. I had no sexual contact that could make me pregnant
   v. I only had sexual contact with someone I was sure didn't have STIs or HIV
   vi. I am infertile or less fertile
   vii. I want to get pregnant or I'm pregnant
   viii. Contraceptives are too expensive
   ix. My sexual partner(s) is infertile or of reduced fertility
   x. My partner(s) uses a contraceptive method
   xi. It is the job of my partner(s) to protect themselves
   xii. My partner(s) does not want us to use contraceptives
   xiii. The use of contraceptives is not accepted in my culture
   xiv. Other, namely:

c. (only if **not** indicated at **5a viii** (condom)): You did <u>not</u> use a condom during your most recent sexual contact, what is the reason for this? You can select multiple answers.
   i. I find it difficult to use condoms correctly
   ii. Condoms are not comfortable
   iii. Condoms are not adapted to my body and needs
   iv. Condoms ruin the atmosphere
   v. Other, namely:

6. Alcohol and drug use during sex

a. **(only if 4c = yes):** Have you consumed alcohol during your sexual contact(s) in the <u>past 6 months</u>?
   i. yes
   ii. no
   iii. I don't know
   iv. I'd rather not say

b. **(only if 4c = yes):** Have you consumed alcohol during your sexual contact(s) in the <u>past 6 months</u>?
   i. yes
   ii. no
   iii. I don't know
   iv. I'd rather not say

c. **(only if 6b = yes)**: Which drugs have you used during sexual contact in the <u>past 6 months</u>? You can select multiple answers.
   i. Cannabis (weed/marijuana)

ii. Cocaine
iii. Amphetamines/speed
iv. Ecstasy/MDMA
v. LSD/Acids
vi. Heroin
vii. Methadone
viii. Morphine plaster
ix. Poppers
x. Other, namely: (text box)

7. Sex work

a. Have you ever done sex work? By 'sex work' we mean whether you have engaged in sex for payment or in exchange for goods.
   i. Yes
   ii. No
   iii. Prefer not to say

b. (only if **7a = yes**): Have you done sex work in the last 6 months?
   i. no
   ii. one time
   iii. about once a month
   iv. two to three times a month
   v. weekly
   vi. daily

f. (only if **7a=yes**): What kind of sex work have you done in the last 6 months?
   i. Window prostitution
   ii. Bar prostitution
   iii. Escort
   iv. Webcam sex
   v. Street prostitution/soliciting
   vi. Private reception (at home)
   vii. Pornography
   viii. Other, namely:

g. (if **7a = yes**): What are or were the main reasons for you to do sex work? Please indicate all answers that apply to you.
   i. Out of financial necessity (lack of food)
   ii. In exchange for goods (e.g. mobile phone)
   iii. In exchange for narcotics (drugs)
   iv. In exchange for hormonal preparations
   v. To save for surgery
   vi. Because I prefer sex work over other work
   vii. Because my gender identity/gender role is confirmed in sex work

viii. Due to no access to the regular labour market (e.g. due to status)
ix. Because of the social support from other sex workers
x. Because it's fun to offer sex
xi. I am/was forced to do this
xii. I am accepted for who I am as a sex worker
xiii. Other, namely:

h. (if **7a = yes**): How often have you used condoms during sex work in the past 6 months?
i. always
ii. often
iii. sometimes
iv. rarely
v. never

i. (if **7a = yes**): What does/does condom use depend on/not depend on?
xi. the client
xii. the pay
xiii. the action
xiv. other, namely:

8. Information and needs

a. Have you ever been given information about sexual health, sexually transmitted infections (STIs) and HIV? You can select multiple answers.
i. Yes, through sex education at school
ii. Yes, through a healthcare provider
iii. Yes, through an LGBTQI+ organization (holebi and/or transgender organization)
iv. Yes, via the internet
v. Yes, I asked friends/acquaintances for information
vi. No, I don't need this
vii. No, but I would like more information

b. Do you feel that the information you now have is applicable or useful to your gender identity and body?
i. Yes
ii. No

c. Can you explain a bit more about this? (Open question)

d. What would you like more information about? You can indicate different options.
i. How to flirt or date someone
ii. Different methods to have safe sexual contact
iii. How HIV is transmitted
iv. How other sexually transmitted infections (STIs) are transmitted
v. The current situation for people with HIV
vi. How to talk/communicate about sex with your partner(s)

vii. How to talk about contraception and/or condom use with your sex partner(s)
viii. How to prevent unwanted pregnancy
ix. How to find someone to have a sexual relationship with
x. How to make a sexual relationship work well
xi. Other, namely:

9. Closing and contact

a. Is there anything you would like to say about this questionnaire? (open question, sufficient space provided)

b. May we contact you for a possible follow-up study?
   i. yes
   ii. no

c. Would you like to be kept informed of the results of this survey by email?
   i. yes
   ii. no

Thank you for your participation! You can follow the progress of the study via https://transgenderinfo.be/l/test-and-tell
