# Supplementary material for: Prevalence and associated risk factors of HIV infections in a representative transgender and non-binary population in Flanders and Brussels (Belgium): Protocol for a community-based, cross-sectional study using time-location sampling
Source: PLoS One. 2022 Apr 11;17(4):e0266078. doi: 10.1371/journal.pone.0266078 (PMC9000107; doi:10.1371/journal.pone.0266078)
Supplement: S1 File — (DOCX) [file pone.0266078.s004.docx]

| Document CPROSPECTIEF OBSERVATIONEEL ONDERZOEK | | | |
| --- | --- | --- | --- |
|  | | | |
|  | | | |
| **COMMISSIE VOOR MEDISCHE ETHIEK** | | | |
| **telefoon**  +32 (0)9 332 56 13 \| +32 (0)9 332 33 36 \| +32 (0)9 332 68 55 | | **fax**  +32 (0)9 332 49 62 | **e-mail**  ethisch.comite@uzgent.be |
|  |  | | |

**VERZOEK TOT ADVIES VAN DE COMMISSIE VOOR MEDISCHE ETHIEK OVER EEN PROSPECTIEF OBSERVATIONEEL ONDERZOEKSPROJECT OP GEZONDHEIDSGEGEVENS**

**Enkel patiëntengegevens verzamelen die klinische standaardgegevens zijn of vragenlijsten / interview afnemen.**

**(= geen enkel aanvullend onderzoek, bloed- of andere staalafname)**

1. Titel van het onderzoek

Het in kaart brengen van transgender gemeenschappen in Vlaanderen

1. Gegevens van de onderzoeker(s)
   [de eerste onderzoeker moet een persoon zijn die vast verbonden is aan de dienst (geen ASO) of universiteit]

Naam: T’Sjoen Voornaam: Guy

Functie: diensthoofd

UZ-dienst: dienst Endocrinologie: Centrum voor Seksuologie en Gender

of faculteit/vakgroep: vakgroep inwendige ziekten

Telefoon/gsm: 093322107

E-mail: guy.tsjoen@uzgent.be

Naam UZ-diensthoofd of vakgroepvoorzitter: prof. dr. Guy T’Sjoen

Naam: Motmans Voornaam: Joz

Functie: Wetenschappelijk medewerker

UZ-dienst: dienst Endocrinologie: Centrum voor Seksuologie en Gender: - Transgender Infopunt

of faculteit/vakgroep: Talen en Letterkunde

Telefoon/gsm: 093321178

E-mail: joz.motmans@uzgent.be

Naam UZ-diensthoofd of vakgroepvoorzitter: prof. dr. Guy T’Sjoen

1. Gegevens van de medewerker(s) aan het onderzoek

Naam: Van Schuylenbergh Voornaam: Judith

Functie: Wetenschappelijk medewerker

UZ-dienst: dienst Endocrinologie: Centrum voor Seksuologie en Gender: - Transgender Infopunt

Telefoon/gsm: 093325725

E-mail: judith.vanschuylenbergh@uzgent.be

Naam UZ-diensthoofd of vakgroepvoorzitter: prof. dr. Guy T’Sjoen

1. Opzet van het onderzoek

Verzameling van patiëntengegevens die klinisch standaardgegevens zijn
(= geen enkel aanvullend onderzoek, bloed- of andere staalafname)

Gegevensverzameling van patiënten door hoofdonderzoeker persoonlijk behandeld

Gegevensverzameling van een groep patiënten op de dienst van de hoofdonderzoeker
met een bepaalde pathologie

Vragenlijsten (gelieve die ook voor te leggen aan het CME)

Interview (Gelieve de vragen ook voor te leggen aan het CME)

1. Is het onderzoek

diagnostisch  therapeutisch

fysiologisch  fysiopathologisch

morfologisch  epidemiologisch

1. Is het onderzoek

monocentrisch

multicentrisch

de Commissie voor Medische Ethiek UZ Gent is de centrale commissie

ja (naam, adres, tel, fax en e-mail van andere Commissies voor Medische Ethiek die

meewerken aan het onderzoek + naam van de lokale onderzoeker)

neen (naam, adres, tel, fax en e-mail van de centrale Commissie voor Medische Ethiek)

1. Wordt dit onderzoeksproject financieel ondersteund?

ja  neen

FWO

BOF

Farmaceutische industrie

Naam:

Andere: Rode Kruis Vlaanderen, Instituut voor de Gelijkheid van Vrouwen en Mannen (IGVM)

1. Wie is de opdrachtgever?

UZ Gent  UGent

Andere: specifieer:

1. Geef een korte samenvatting van het onderzoeksproject (minimum 30 zinnen,
   verstaanbaar voor mensen die niet gespecialiseerd zijn in de materie)

Deze studie is een eerste, voorbereidend onderdeel van een studie naar de prevalentie van HIV bij transgender personen. Internationaal onderzoek wijst immers uit dat transgender personen (zijnde personen die zich niet of in mindere mate identificeren met het geslacht dat hen bij geboorte werd toegekend), een risicogroep zijn voor besmetting met HIV. Europees en Vlaams onderzoek ontbreekt echter volledig. Met deze ‘transgender HIV studie’ willen we de HIV prevalentie bij Vlaamse transgender en non-binaire personen, alsook geassocieerde factoren, in kaart brengen.

Omdat de transgender gemeenschap in Vlaanderen onsamenhangend en verspreid is, zal eerst een ‘community mapping’ studie worden opgezet, met als doel de transgender gemeenschap in Vlaanderen in kaart te brengen, om zo een meer representatieve steekproef te kunnen bekomen voor de eigenlijke HIV prevalentie studie. Binnen deze studie zal onderzocht worden op welke plaatsen transgender personen komen of aanwezig zijn, zowel fysiek (events, bijeenkomsten, wachtzalen, …) als digitaal (forums, facebookgroepen). De huidige EC-aanvraag heeft enkel betrekking op deze voorbereidende studie, die het design van de eigenlijke HIV studie moet informeren.

De studie zal gebruik maken van een etnografische methode, waarbij zowel participerende observatie, informele gesprekken als diepte-interviews met sleutelinformanten worden gebruikt om community settings van de transgender gemeenschap in Vlaanderen in kaart te brengen. Sleutelinformanten kunnen zowel transgender of non-binaire personen zelf zijn als hulpverleners met expertise over deze doelgroep. De diepte-interviews kunnen zowel face-to-face als digitaal worden afgenomen, afhankelijk van de voorkeur van de participant en de op dat moment geldende COVID maatregelen.

Participerende observatie is het bestuderen van onderzoeksobjecten in hun natuurlijke setting en houdt in dat de onderzoeker aanwezig is binnen een bepaalde setting, en deze setting door observatie en informele gesprekken met aanwezigen in kaart brengt. Voorbeelden van settings zijn cafés, praatgroep bijeenkomsten, feestjes. Voor participerende observatie en informele gesprekken worden veldnota’s gebruikt om data te verzamelen. Hierbij worden geen persoonsgegevens genoteerd, enkel kenmerken van de setting.

De diepte-interviews worden opgenomen en getranscribeerd, mits toestemming van de participant, en digitaal bewaard op de UZ servers. Audiobestanden worden slechts bewaard voor de duurtijd van het onderzoek en nadien verwijderd. Alle data zal worden gepseudonimiseerd volgens een geautomatiseerd systeem. Transcripten worden bewaard onder het pseudoniem van de participant en persoonsgegevens worden enkel genoteerd en gebruikt om in functie van de snowball sampling nieuwe respondenten te contacteren. In geval van online diepte-interviews wordt het Informed Consent formulier vooraf doorgemaild en wordt aan de participant gevraagd voor de start van het interview een ondertekend exemplaar terug te sturen. Dataverzameling vindt plaats tot saturatie wordt bereikt. De topiclijst voor de diepte-interviews is te raadplegen in bijlage.

Deze studie zal resulteren in een overzicht van community settings waarbinnen transgender personen in Vlaanderen aanwezig zijn, de kenmerken van elke setting en van de bezoekers ervan. Deze voorbereidende studie zal de basis vormen voor het bepalen van de steekproef van de eigenlijke HIV studie, waarbij we gebruik zullen maken van een 2-stage Time Location Sampling frame. Op basis van de community mapping analyse zal een vooraf bepaald aantal settings en tijdsloten random worden geselecteerd voor recrutering. Binnen de geselecteerde settings en tijdsloten zal een vooraf bepaald aantal respondenten random worden geselecteerd voor deelname aan de HIV studie.

Voor de HIV studie, die zal gebruik maken van speekseltesten om de HIV prevalentie binnen deze populatie te schatten, zal afzonderlijk ethische goedkeuring worden aangevraagd.

1. Gegevens over

Volwassenen in staat om toestemming te geven

Volwassenen niet in staat om toestemming te geven

Minderjarigen

Studie in urgentiesituatie

1. De deelnemers zijn

gezonden

zieken

lijdend aan:

personeel, studenten

geslacht

man

vrouw

x

1. Hoe worden deze deelnemers gerekruteerd?

**Opgelet: het experiment is enkel verzekerd voor het aantal dat hier is opgegeven.**

**Indien men extra deelnemers wil includeren, zal men dat via een amendement moeten aanvragen.**

Rekrutering gebeurt via snowball sampling: bij de start worden 5 sleutelinformanten gecontacteerd binnen het netwerk van het Transgender Infopunt, die wegens hun positie expertise hebben over (een deel van) de transgender gemeenschap in Vlaanderen. Zij kunnen de onderzoeker introduceren in bepaalde groepen en/of doorsturen naar andere relevante sleutelinformanten. Een diverse groep sleutelinformanten wordt gekozen in termen van genderidentiteit, seksuele voorkeur, etniciteit, leeftijd etc.

Er wordt hierbij geen patiëntendata gebruikt. Er is geen rekruteringsmateriaal dat gebruikt wordt om een algemene oproep tot deelname te doen, gezien sleutelinformanten strategisch gekozen worden op basis van hun positie in de transgender gemeenschap en persoonlijk worden aangesproken door de onderzoeker.

1. Totaal aantal deelnemers in de studie?

In het UZ Gent:

Extern in België: 50

1. Verzekering

Door welke verzekeringspolis bent u gedekt? UZ Gent-no fault

(wanneer de verzekering niet is afgesloten door UZ Gent/UGent, gelieve dan de verzekeringspolis bij te voegen)

Datum: Klik of tik om een datum in te voeren.

1. Einddatum experiment

Datum: 31 december 2020

**Let wel op: elk experiment op mensen na de einddatum is niet meer gedekt door de verzekering**

**zodat u op dat ogenblik in overtreding bent met de wettelijke beschikkingen.**

**U kan een verlenging van het experiment bij de commissie voor medische ethiek aanvragen.**

1. Wordt voor dit onderzoek een informed consent gevraagd aan de patiënt/wettelijke vertegenwoordiger voor inzage van dossier, afname vragenlijsten/interview
   (steeds het IC ter goedkeuring aan het CME voor leggen)?

ja

neen (Argumenteren waarom niet)

Voor participerende observatie zal steeds vooraf toestemming worden gevraagd aan de organisator of verantwoordelijke om aanwezig te zijn binnen de gekozen setting (evenement, bijeenkomst). De mogelijkheid tot aanwezig zijn binnen deze settings is afhankelijk van en steeds conform de op dat moment geldende COVID maatregelen.

Voor informele gesprekken wordt mondelinge informed consent gevraagd. De onderzoeker legt hierbij eerst het doel van de studie uit en vraagt aan de participant of die enkele vragen mag stellen over de transgender gemeenschap waar deze persoon deel van uitmaakt of kennis over heeft. De onderzoeker maakt duidelijk dat er op geen enkele manier identificeerbare informatie zal worden gebruikt.

Voor diepte-interviews wordt een informed consent formulier gebruikt om geïnformeerde toestemming te vragen aan participanten (zie bijlage).

Elke mogelijke participant ontvangt bij een gesprek met de onderzoeker een kaartje met informatie over de studie, een link naar de HIV studie website en de contactgegevens van de onderzoeker.

**Indien neen, zal de Commissie voor Medische Ethiek dat bespreken en als het aanvaardbaar is, aan**

**de onderzoekers vragen de informatie- en waarschuwingsnota over de verwerking van informatie**

**voor wetenschappelijk onderzoek te ondertekenen (zie bijlage).**

**Ik verklaar de gehele verantwoordelijkheid van het hierboven vermeld project op mij te nemen en bevestig dat, voor zover de huidige kennis het toelaat, de inlichtingen met de werkelijkheid overeenstemmen.**

| De hoofdonderzoeker  **datum**  **naam**  **handtekening** |  | Het UZ-diensthoofd of de vakgroepvoorzitter  (voor akkoord)  **datum**  **naam**  **handtekening** |
| --- | --- | --- |
| **Medewerkers aan het onderzoek** |  |  |
| **datum**  **naam**  **handtekening** |  |  |
