# Supplementary material for: Prevalence and associated risk factors of HIV infections in a representative transgender and non-binary population in Flanders and Brussels (Belgium): Protocol for a community-based, cross-sectional study using time-location sampling
Source: PLoS One. 2022 Apr 11;17(4):e0266078. doi: 10.1371/journal.pone.0266078 (PMC9000107; doi:10.1371/journal.pone.0266078)
Supplement: S2 File — (DOCX) [file pone.0266078.s005.docx]

| Document CPROSPECTIVE OBSERVATIONAL RESEARCH | | | |
| --- | --- | --- | --- |
|  | | | |
| **MEDICAL ETHICS COMMITTEE** | | | |
| **phone**  +32 (0)9 332 56 13 \| +32 (0)9 332 33 36 \| +32 (0)9 332 68 55 | | **fax**  +32 (0)9 332 49 62 | **e-mail**  ethisch.comite@uzgent.be |
|  |  | | |

**REQUEST FOR ADVICE FROM THE MEDICAL ETHICS COMMITTEE ON PROSPECTIVE OBSERVATIONAL RESEARCH PROJECT RELATED TO HEALTH DATA**

**Only collect patient data that are clinical standard data or conduct questionnaires/interview.**

**(= no additional research, blood or other sample collection)**



1. **Title of the research project**

Mapping transgender communities in Flanders

1. **Data of the researcher(s)
   [the first researcher must be a person permanently attached to the service (no ASO) or university]**

Name: T'Sjoen First name: Guy

Position: Full professor and head

Center: Department of Endocrinology: Center for Sexology and Gender

Faculty/department: Faculty of Medicine and Health Sciences - Department of Internal Medicine and Pediatrics

Telephone/mobile phone: 093322107

E-mail: guy.tsjoen@uzgent.be

Name head of department or department chair: Prof. Dr. Guy T'Sjoen

Name: Motmans First name: Joz

Position: Scientific collaborator

Center: Department of Endocrinology: Center for Sexology and Gender: Transgender Infopunt

Faculty/department: Faculty of Arts and Philosophy - Department of Languages and cultures

Telephone/mobile phone: 093321178

E-mail: joz.motmans@uzgent.be

Name head of department or department chair: Prof. Dr. Guy T'Sjoen

1. **Details of the employee(s) involved in the study**

Name: Van Schuylenbergh First name: Judith

Position: Scientific collaborator

Position: Scientific collaborator

Center: Department of Endocrinology: Center for Sexology and Gender: Transgender Infopunt

Telephone/mobile phone: 093325725

E-mail: judith.vanschuylenbergh@uzgent.be

Name UZ head of department or department chair: Prof. Dr. Guy T'Sjoen

1. **Design of the study**

☐ Collection of patient data that are clinically standard data
(= no additional examinations, blood or other sampling)

☐ Data collection of patients treated personally by the principal investigator

☐ Data collection of a group of patients on the principal investigator's service
with a particular pathology

☐ Questionnaires (please also submit these to the CME)

☒ Interview (please also submit the questions to the CME)

1. **Is the research**

☐ diagnostic ☐ therapeutic

☐ physiological ☐ physiopathological

☐ morphological ☒ epidemiological

1. **Is the research**

☒ monocentric

☐ multicentric

☐ the Commission for Medical Ethics UZ Gent is the central committee

☐ yes (name, address, tel, fax and e-ma il of other Medical Ethics Committees

participating in the study + name of the local investigator)

☐ no (name, address, tel, fax and e-mail of the Central Committee on Medical Ethics)

1. **Is this research project financially supported?**

☒ yes ☐ no

☐ FWO

☐ BOF

☐ Pharmaceutical industry

Name:

☒ Other: Belgian Red Cross-Flanders, Institute for the Equality of Women and Men (IGVM)

1. **Who is the sponsor of the study?**

☒ UZ Gent ☐ UGent

☐ Other: specify:

1. **Provide a short summary of the protocol (minimum 30 sentences/one half page and maximum one page), intelligible to people not specialized in the matter. Do not just refer to an attached protocol.**

This study is a first, preparatory part of a study on the prevalence of HIV in transgender persons. International research has shown that transgender people (being people who do not identify, or to a lesser extent, identify with the sex assigned to them at birth) are a high-risk group for becoming infected with HIV. However, European and Flemish research is completely lacking. With this 'transgender HIV study' we want to map the HIV prevalence in Flemish transgender and non-binary persons, as well as associated factors.

Because the transgender community in Flanders is incoherent and scattered, a community mapping study will first be executed. This preparatory study aims to map the transgender community in Flanders in order to obtain a representative sample for the HIV prevalence study in the next phase of the project. Within this study, it will be investigated at which places transgender people gather, both physically (events, meetings, waiting rooms, ...) and digitally (forums, Facebook groups). The current EC application relates only to this preliminary study, which should inform the design of the actual HIV study.

The study will use an ethnographic method, in which participatory observation, informal conversations and in-depth interviews with key informants are used to map community settings of the transgender community in Flanders. Key informants can be transgender or non-binary persons themselves as well as care providers with expertise with this target group. The in-depth interviews can be conducted face-to-face or digitally, depending on the preference of the participant and the current COVID-19 measures.

Participatory observation is the study of research objects in their natural setting and means that the researcher is present within a certain setting, and maps this setting through observation and informal conversations with those present. Examples of settings are bars, support group meetings or parties. Field notes are used to collect data for participant observation and informal conversations. No personal data is collected here, only characteristics of the setting.

The in-depth interviews are recorded and transcribed, with the permission of the participant, and digitally stored on the hospital servers. Audio files are only kept for the duration of the study and then deleted. All data will be pseudonymised according to an automated system. Transcripts are kept under the pseudonym of the participant and personal data are only noted and used to contact new respondents in function of snowball sampling. In the case of online in-depth interviews, the Informed Consent form is emailed in advance and the participant is asked to return a signed copy before the start of the interview. Data collection takes place until saturation is reached. The topic list for the in-depth interviews can be found in the appendix.

This study will result in an overview of community settings in which transgender people gather in Flanders, the characteristics of each setting and of its visitors. This preliminary study will form the basis for developing a sampling framework for the actual HIV prevalence study, using a 2-stage Time Location Sampling strategy. Based on the community mapping analysis, a predetermined number of settings and time slots will be randomly selected for participant recruitment. Within the selected settings and time slots, a predetermined number of respondents will be randomly selected to participate in the HIV study.

The HIV prevalence study, which will use saliva testing to estimate HIV prevalence in this population, will be subjected to a separate ethical approval.

1. **Data on**

☒ Adults able to give consent

☐ Adults unable to give consent

☐ Minors

☐ Study in emergency situation

1. **The participants were**

☒ healthy

☐ sick

suffering from:

☐ staff, students

☒ gender

☒ male

☒ female

☒ x

1. **How are these participants recruited?**

**Attention: the experiment is only insured for the amount specified here.**

**If you want to include additional participants, you will have to request this via an amendment.**

Snowball sampling will be used for selecting participants: at the start, 5 key informants are contacted within the network of the Transgender Infopunt. Transgender Infopunt has expertise on (part of) the transgender community in Flanders. They can introduce the researcher to certain groups and/or forward them to other relevant key informants. A diverse group of key informants is chosen in terms of gender identity, sexual preference, ethnicity, age, etc.

No patient data are used here. There is no recruitment material used to make a general call for participation, as key informants are strategically chosen based on their position in the transgender community and are personally contacted by the researcher.

1. **Total number of participants in the study?**

☐ At UZ Gent:

☒ Externally in Belgium: 50

1. **Insurance**

Which insurance policy covers you? UZ Gent-no fault

(if the insurance has not been taken out by UZ Gent/UGent, please enclose the insurance policy)

Date: Click or tap to enter a date.

1. **End date of experiment**

Date: 31 December 2020

**Please note: any experiment on humans after the end date is no longer covered by the insurance,**

**so that you are in violation of the legal provisions at that time.**

**You can request an extension of the experiment from the Medical Ethics Committee.**

1. **For this research, is informed consent requested from the patient/legal representative for inspection of the file, taking questionnaires/interview
   (always submit the IC to the CME for approval)?**

☒ yes

☐ no (Arguing why not)

For participant observation, prior permission will always be requested from the organizer or responsible person to be present within the chosen setting (event, meeting). The possibility of being present within these settings depends on the current COVID-19 measures.

For informal conversations, oral informed consent is requested. The researcher first explains the purpose of the study and asks the participant if he or she may ask some questions about the transgender community that this person is part of or has knowledge about. The researcher makes it clear that no identifiable information will be used in any way.

For in-depth interviews, an informed consent form is requested from participants (see appendix).

Each potential participant will receive a card with information about the study, a link to the HIV study website and the contact details of the researcher.



**I declare that I take full responsibility for the project mentioned above and confirm that, to the best of current knowledge, the information corresponds to reality.**

| **The principal investigator**  **date**  ****  **name**  ****  **signature**  **** |  | **The UZ department head or department chair**  **(for approval)**  **date**  ****  **name**  ****  **signature**  **** |
| --- | --- | --- |
| **Research staff** |  |  |
| **date**  ****  **name**  ****  **signature**  **** |  |  |
