# Supplementary material for: Prevalence and associated risk factors of HIV infections in a representative transgender and non-binary population in Flanders and Brussels (Belgium): Protocol for a community-based, cross-sectional study using time-location sampling
Source: PLoS One. 2022 Apr 11;17(4):e0266078. doi: 10.1371/journal.pone.0266078 (PMC9000107; doi:10.1371/journal.pone.0266078)
Supplement: S3 File — (DOCX) [file pone.0266078.s006.docx]

| Document AINTERVENTIONEEL ACADEMISCH ONDERZOEK | | | |
| --- | --- | --- | --- |
|  | | | |
|  | | | |
| **COMMISSIE VOOR MEDISCHE ETHIEK** | | | |
| **telefoon**  +32 (0)9 332 56 13 \| +32 (0)9 332 33 36 \| +32 (0)9 332 68 55 | | **fax**  +32 (0)9 332 49 62 | **e-mail**  ethisch.comite@uzgent.be |
|  |  | | |

**VERZOEK TOT ADVIES VAN DE COMMISSIE VOOR MEDISCHE ETHIEK OVER EEN ONDERZOEKS­PROJECT BIJ DE MENS**

**EUDRACT NUMMER** (indien interventioneel geneesmiddelenonderzoek): NVT

1. Titel van het onderzoek

**Prevalence and associated risk factors of HIV infections in a representative transgender and non-binary population in Flanders (Belgium): a community-based, cross-sectional study using time-location sampling**

1. Gegevens van de onderzoeker(s)
   [de eerste onderzoeker moet een persoon zijn die vast verbonden is aan de dienst (geen ASO) of universiteit]

Naam: T’Sjoen Voornaam: Guy

Functie: diensthoofd

UZ-dienst: dienst Endocrinologie: Centrum voor Seksuologie en Gender

of faculteit/vakgroep: Faculteit Geneeskunde en Gezondheidswetenschappen - Vakgroep Inwendige ziekten en Pediatrie

Telefoon/gsm: 093322107

E-mail: guy.tsjoen@uzgent.be

Naam UZ-diensthoofd of vakgroepvoorzitter: prof. dr. Guy T’Sjoen

Naam: Motmans Voornaam: Joz

Functie: Wetenschappelijk medewerker

UZ-dienst: dienst Endocrinologie: Centrum voor Seksuologie en Gender: Transgender Infopunt

of faculteit/vakgroep:

Telefoon/gsm: 093321178

E-mail: joz.motmans@uzgent.be

Naam UZ-diensthoofd of vakgroepvoorzitter: prof. dr. Guy T’Sjoen

Gegevens van de medewerker(s) aan de studie

Naam: Van Schuylenbergh Voornaam: Judith

Functie: Wetenschappelijk medewerker

UZ-dienst: dienst Endocrinologie: Centrum voor Seksuologie en Gender: Transgender Infopunt

Telefoon/gsm: 093325725

E-mail: judith.vanschuylenbergh@uzgent.be

Naam UZ-diensthoofd of vakgroepvoorzitter: prof. dr. Guy T’Sjoen

1. Soort onderzoek

interventioneel onderzoek

met geneesmiddel (alle items van toepassing aanduiden)

fase I

fase II

fase III

fase IV

proef voor gentherapie en somatische celtherapie

proef met geneesmiddelen die genetisch gewijzigde organismen bevatten

proef met celtherapie met xenogenen

andere

medical device

bloedafname, RX, …

andere: speekselstaalafname en vragenlijsten

1. Is het onderzoek

diagnostisch  therapeutisch

fysiologisch  fysiopathologisch

morfologisch  epidemiologisch

1. Is het onderzoek in België

monocentrisch

multicentrisch

de Commissie voor Medische Ethiek UZ Gent is de centrale commissie

ja (naam, adres, tel, fax en e-mail van andere Commissies voor Medische Ethiek die

meewerken aan het onderzoek + naam van de lokale onderzoeker)

neen (naam, adres, tel, fax en e-mail van de centrale Commissie voor Medische Ethiek)

1. Gaat dit onderzoek ook door in het buitenland NVT

in Europa – Welke zijn de deelnemende landen:

in de Verenigde Staten

1. Wordt deze studie financieel ondersteund?

ja  neen

FWO/BOF

farmaceutische industrie:

andere: Rode Kruis Vlaanderen, Instituut voor de Gelijkheid van Vrouwen en Mannen (IGVM), Gilead (zie bijlage 6, 7 en 8)

1. Wie is de opdrachtgever van de studie die niet door de industrie wordt gesponsord?

medewerker van het UZ Gent (naam en adres): T’Sjoen Guy

medewerker van de UGent (naam en adres):

andere, specifieer (naam en adres):

1. Geef een korte samenvatting van het protocol (minimum 30 zinnen/ een halve pagina en maximum één pagina), verstaanbaar voor mensen niet gespecialiseerd in de materie. Verwijs niet alleen naar een bijgevoegd protocol.

Internationaal onderzoek wijst uit dat transgender personen een risicogroep zijn voor besmetting met HIV. Europees en Vlaams onderzoek ontbreekt echter volledig. Dit terwijl regionaal beleid, zoals het bloeddonatiebeleid van Rode Kruis Vlaanderen (mede-financierder van deze studie), dat momenteel transgender personen uitsluit voor bloeddonatie, zich op niet-Belgische en zelfs niet-Europese HIV prevalentiecijfers baseert. Gezien er geen wetenschappelijke basis is voor dergelijk uitsluitingsbeleid in Vlaanderen, kan dit worden beschouwd als discriminatie op basis van genderidentiteit. Met deze studie willen we de HIV prevalentie bij Vlaamse transgender en non-binaire personen, en geassocieerde factoren, in kaart brengen.

Omdat de transgender gemeenschap in Vlaanderen onsamenhangend en verspreid is, werd eerst een **voorbereidende studie** opgezet waarin transgender gemeenschappen in kaart werden gebracht (zie EC-aanvraag BC-08157). Deze studie vormt de basis voor het sampling frame van de HIV studie, zodat een meer representatieve steekproef kan bekomen worden. De HIV studie maakt gebruik van een 2-stage Time Location Sampling (TLS) frame: van het totaal aantal settings dat werd in kaart gebracht wordt een aantal settings random geselecteerd; waarna binnen elke geselecteerde setting een bepaald aantal respondenten random wordt geselecteerd voor deelname aan de studie. Settings kunnen zowel fysieke settings zijn (bijvoorbeeld: evenementen, cafés, praatgroep bijeenkomsten, praktijk van hulpverleners) als online settings (bijvoorbeeld: facebookgroepen, Discord-fora).

Aan de hand van **speekseltesten** (en indien reactief confirmatie met een bloedtest indien mogelijk) zal de HIV prevalentie binnen de transgender populatie worden geschat. De studie wordt uitgevoerd in samenwerking met het Instituut voor Tropische Geneeskunde (ITG) en maakt gebruik van het online platform [www.swab2know.eu](http://www.swab2know.eu), dat reeds wordt gebruikt voor een gelijkaardige studie naar HIV bij Mannen die seks hebben met Mannen (MSM). Deelnemers dienen, na het doorlopen van een informatiepagina en aanklikken van toestemming (zie bijlage 3 en 4), eerst te registreren met een e-mailadres (zie verder), waarna ze een korte vragenlijst dienen in te vullen (zie bijlage 1) en gevraagd worden zelf een speekselstaal af te nemen.

De studie wordt uitgedragen/uitgevoerd door het Transgender Infopunt (UZ Gent) omdat dit een gekend en vertrouwd informatie- en expertisecentrum is bij de doelpopulatie. Een studiespecifieke pagina zal worden uitgewerkt op de website [www.transgenderinfo.be](http://www.transgenderinfo.be), waar men extra kadering van de studie zal kunnen vinden (zie bijlage 5). Deelnemers worden zowel in fysieke als online settings gerekruteerd.

Dataverzameling binnen fysieke settings verloopt via een tablet waarop de deelnemer zelf op het swab2know platform de vragenlijst doorloopt. De deelnemer neemt zelf een speekselstaal af en bezorgt dit aan de onderzoeker. De onderzoeker verzendt binnen de 5 dagen de verzamelde speekselstalen naar het ITG, waar de stalen worden verwerkt en geanalyseerd (zie bijlage 9). Binnen grote fysieke settings (feestjes, evenementen) of moeilijker te bereiken delen van de doelpopulatie (bijvoorbeeld: personen van kleur, sekswerkers) is het mogelijk dat de onderzoeker voor de dataverzameling wordt bijgestaan door een *peer recruiter* of sleutelfiguur binnen de transgender community die de studie mee uitdraagt en in sommige gevallen het vertrouwen in de studie dient te vergroten.

Online dataverzameling verloopt rechtstreeks via het platform [www.swab2know.eu](http://www.swab2know.eu). Deelnemers krijgen een speekseltest opgestuurd en dienen deze te verzenden naar het ITG in een voorgefrankeerde enveloppe.

Inclusiecriteria voor deelname zijn: meerderjarig zijn, identificeren als transgender of non-binair (zelf-identificatie) en wonen in Vlaanderen of Brussel.

De studie heeft als voordeel voor deelnemers dat zij enkele weken na **deelname het resultaat van de HIV test kunnen opvragen**. Daarom dienen ze te registreren met een e-mailadres. De medewerker van het ITG voert de resultaten in, waarna deelnemers een e-mail ontvangen met de melding dat zij hun resultaat kunnen bekijken via het swab2know platform. Deelnemers met een reactief resultaat worden doorverwezen naar hun huisarts of een arts naar keuzevoor een confirmatietest via bloedafname. Het ITG zorgt voor opvolging en linkage to care: doorverwijzing maakt deel uit van de standard routine care en is geen onderdeel van de studie, maar ITG neemt wel een faciliterende rol op. Zie ook: <https://www.swab2know.eu/BE/N/hoewerkthet/>

Deelnemers krijgen 2 weken na afname van het speekselstaal via e-mail de vraag tot deelname aan een vervolgvragenlijst (zie bijlage 2), indien ze toestemming gaven om hiervoor gecontacteerd te worden.

Alle gegevens worden gepseudonimiseerd en gekoppeld via een codesysteem, waartoe enkel de hoofdonderzoeker en de medewerker van het ITG die de enveloppen verzendt,de testresultaten ingeeft en de communicatie naar de deelnemer opneemt toegang krijgen. Alle andere onderzoekers krijgen enkel toegang tot een gepseudonimiseerd databestand. De speekselstalen worden gecodeerd geanalyseerd.

De verworven data zal worden geanalyseerd door de onderzoeker verbonden aan het UZ Gent (Judith Van Schuylenbergh) en de onderzoeker verbonden aan het Center for Evidence Based Practice (CEBAP) van het Rode Kruis Vlaanderen (NDB). De resultaten van de studie zullen zowel worden gerapporteerd in een breed toegankelijk rapport in opdracht van het Instituut voor de Gelijkheid van Vrouwen en Mannen (IGVM) als in wetenschappelijke artikels.

Een uitgebreid draft protocol van deze studie vindt u in bijlage 10.

1. Wat zijn de argumenten (theoretische, experimentele of andere) die een voordeel laten verwachten van de te testen nieuwe methode, preparaat, … boven de bekende en reeds gebruikte?

Internationaal onderzoek wijst op een zeer hoge prevalentie van HIV onder transgender personen, maar Europees onderzoek binnen deze thematiek ontbreekt echter compleet. Het merendeel van het bestaande onderzoek werd uitgevoerd in de grootstedelijke context van San Francisco of New York, of Aziatische landen waar de maatschappelijke context voor transgender personen sterk verschilt van de Europese context. Bovendien is het bestaande onderzoek beperkt door een selectieve focus op bepaalde delen van de transgender populatie (transgender vrouwen, sekswerkers) en worden er vaak convenience sample methoden gebruikt die zorgen voor samples met disproportioneel veel hoog-risico personen (sexual health clinics, community centers, prostitutiebuurten). (Zie ook Van Schuylenbergh, Motmans & Coene, 2018).

De Wereldgezondheidsorganisatie (WHO) baseert zich op dit selectief en niet-representatieve onderzoek voor hun richtlijnen voor preventie en behandeling van HIV binnen 5 sleutelpopulaties: mannen die seks hebben met mannen (MSM), personen die drugs injecteren, personen in gesloten instellingen, sekswerkers en transgender personen (WHO, 2014). Ook het Rode Kruis Vlaanderen baseert zich op dit onderzoek om een uitsluiting voor bloeddonatie in te richten voor transgender personen. Concreet gelden vandaag voor bloeddonatie grotendeels dezelfde regels voor transgender personen als voor MSM, waarvoor wel voldoende Vlaams onderzoek bestaat om een (gedeeltelijke) uitsluiting te beargumenteren. Gezien het gebrek aan representatieve data die valide is voor de Vlaamse transgender populatie, kan de uitsluiting van transgender personen echter gezien worden als discriminatie.

Dit onderzoek heeft als doel HIV te testen in een zo representatief mogelijke steekproef van Vlaamse transgender personen. Het zal in eerste instantie direct leiden tot argumentatie om het bloeddonatiebeleid van het Rode Kruis Vlaanderen te onderbouwen. Het Rode Kruis Vlaanderen is daarom samen met het Instituut voor de Gelijkheid van Vrouwen en Mannen (IGVM) een belangrijke partner in dit onderzoek. Anderzijds zal het ook leiden tot een meer genuanceerd prevalentiecijfer van HIV in een breed staal van de transgender populatie, en inzicht geven in verschillende risicogroepen binnen die zeer diverse transgender populatie. Het opzet voor deze studie is uniek binnen het bestaand onderzoek naar HIV bij transgender personen, omwille van de beoogde steekproefgrootte en diversiteit, het gebruik van de TLS methode en het gebruik van laboratorium-geconfirmeerde HIV data via speekseltesten op deze schaal. We hopen hiermee HIV onderzoek bij transgender personen op de Europese onderzoeksagenda te zetten.

Referenties:

 World Health Organisation (2014). [*Consolidated guidelines on HIV prevention, diagnosis, treatment and care for key populations.*](https://www.who.int/hiv/pub/guidelines/keypopulations/en/) Report, WHO.

Van Schuylenbergh, Motmans & Coene (2018). [Transgender and non-binary persons and sexual risk: A critical review of 10 years of research from a feminist intersectional perspective](https://journals.sagepub.com/doi/full/10.1177/0261018317732478). *Critical Social Policy,* 38:2.

1. Werd een analoog onderzoek al elders uitgevoerd, in zijn geheel of gedeeltelijk?

Zo ja, waar? Wat was het resultaat? Waarom wordt het in deze studie hernomen?

Het Instituut voor Tropische Geneeskunde (ITG, Antwerpen), één van de partners binnen het onderzoek, heeft veel ervaring met de methoden die gebruikt zullen worden in het huidige onderzoek. Het ITG gebruikt deze methoden al jaren voor hun onderzoek naar HIV bij enerzijds Mannen die Seks hebben met Mannen (MSM) en anderzijds Sub-Sahara Migranten (SAM). Dit onderzoek betreft doelpopulaties die substantieel verschillen van de transgender populatie die zal onderzocht worden in de voorgestelde studie. Dit onderzoek is zowel vernieuwend binnen de bestaande HIV literatuur als de bestaande literatuur rond de gezondheid van transgender personen.

De Time-Location Sampling (TLS) strategie, het gebruik van peer recruiters/sleutelfiguren en het gebruik van de speekseltesten in combinatie met een vragenlijst via een tablet werd succesvol gebruikt in het ‘Together project’, een studie naar HIV bij Sub-Sahara Migranten (SAM) in Antwerpen (Nöstlinger & Loos, 2016; Loos et al., 2016; Loos et al., 2017).

Het protocol voor de dataverzameling van de huidige studie werd zo goed als volledig overgenomen van de studie ‘Swab2know’ van het ITG. Swab2know is een HIV-preventieproject gericht op Mannen die Seks hebben met Mannen (MSM). Binnen deze studie wordt gebruik gemaakt van het platform [www.swab2know.eu](http://www.swab2know.eu) waarop personen gratis en anoniem een HIV speekseltest kunnen aanvragen. De onderzoekers trekken daarnaast op bepaalde momenten ’s avonds en in het weekend naar clubs, sauna’s en feestjes om gratis en anonieme speekseltesten aan te bieden. De gebruikte methode werd effectief bevonden en werd positief onthaald binnen de oorspronkelijke doelpopulatie van MSM (Platteau et al., 2015). De studie ‘Swab2know’ is voornamelijk gericht op HIV preventie (verhogen van het testgedrag) en naast de verschillende doelpopulatie is dit het grootste verschil met de huidige studie, die gericht is op het in kaart brengen van de HIV prevalentie binnen een tot nu toe niet bestudeerde populatie in Vlaanderen.

Referenties:

Loos, J., Nöstlinger, C., Vuylsteke, B., Deblonde, J., Ndungu, M., Kint, I., . . . Laga, M. (2017). First HIV prevalence estimates of a representative sample of adult sub-Saharan African migrants in a European city. Results of a community-based, cross-sectional study in Antwerp, Belgium. *PloS one*, 12(4), e0174677.

Loos, J., Vuylsteke, B., Manirankunda, L., Deblonde, J., Kint, I., Namanya, F., . . . Adobea, D. (2016). Together project to increase understanding of the HIV epidemic among Sub-Saharan African migrants: Protocol of community-based participatory mixed-method studies. *JMIR research protocols*, 5(1), e48.

Nöstlinger, C., & Loos, J. (2016). Involving lay community researchers in epidemiological research: experiences from a seroprevalence study among sub-Saharan African migrants. *AIDS care,* 28(sup1), 119-123.

Platteau, T., Fransen, K., Apers, L., Kenyon, C., Albers, L., Vermoesen, T., ... & Florence, E. (2015). Swab2know: an HIV-testing strategy using oral fluid samples and online communication of test results for men who have sex with men in Belgium. *Journal of medical Internet research*, *17*(9), e213.

1. Zal een chemische substantie toegediend worden?

ja  neen

Zo ja:

a. Langs welke weg?

b. Naam en oorsprong van de substantie:

c. Aan wie wordt de receptie, opslag, verdeling en terugsturen van niet-gebruikte chemische

substanties toevertrouwd?

d. Zullen radio-isotopen toegediend worden?

ja  neen

Welke?

1. Indien het om een nieuwe substantie gaat: heeft de onderzoeker kennisgenomen van het volledige toxicologische, dierfarmacologische en humane dossier? NVT

ja  neen

Zo neen, leg uit:

1. Keuze van de proefpersonen:

a. Gezonden?

ja  neen

Patiënten lijdend aan:

b. Zwangere vrouwen of vrouwen die tijdens het onderzoek zwanger kunnen worden?

ja  neen

c. Aantal proefpersonen in het UZ Gent: 800

d. Aantal proefpersonen extern (in België): 600

Aantal proefpersonen binnen België = 800 + 600 = 1400

**Opgelet: het experiment is enkel verzekerd voor het aantal dat hier opgegeven wordt.**

**Indien men extra deelnemers wil includeren, zal men dat via een amendement moeten aanvragen.**

e. Leeftijd: meerderjarigen (18+)

f. Geslacht: m/v/x

g. Hoe worden ze gerekruteerd?

Deelnemers binnen niet-klinische fysieke settings worden aangesproken door de onderzoeker, die de inclusiecriteria (18 jaar of ouder, identificeren als transgender of non-binair en wonen in Vlaanderen of Brussel) overloopt en het doel van de studie uitlegt. Na mondelinge toestemming wordt de deelnemer gevraagd de stappen op het swab2know platform te doorlopen op de tablet. Hier krijgen deelnemers eerst nogmaals alle informatie en het informed constent form te lezen, waarna men digitaal nogmaals toestemming moet geven om door te gaan naar de vragenlijst en instructies voor afname van de speekseltest.

Na afloop krijgen deelnemers een kaartje met contactgegevens van het onderzoeksteam, een link en QRcode naar de studiespecifieke pagina’s op de website [www.transgenderinfo.be](http://www.transgenderinfo.be), waar alle informatie rond de studie te vinden is en waar updates zullen worden geplaatst over het onderzoek en de resultaten.

Deelnemers die online worden geworven, worden aangesproken door de onderzoeker of peer recruiter, die hen de link van het swab2know platform doorstuurt, waarop ze zelf de stappen kunnen doorlopen en de speekseltest thuis krijgen opgestuurd. Zij worden na afloop van de studie ook doorgestuurd naar de studiespecifieke pagina’s op de website [www.transgenderinfo.be](http://www.transgenderinfo.be).

Deelnemers krijgen in de vragenlijst de optie om contactgegevens te noteren om op de hoogte gehouden te worden over het verloop en de resultaten van de studie. Indien deelnemers hier toestemming voor geven zullen zij gedurende het project enkele malen een update ontvangen via email (maximum 5 mailings).

**Er worden geen patiëntengegevens gebruikt. Deelnemers zijn niet noodzakelijk patiënten van het UZ Gent.**

Binnen het UZ Gent vormen de consultaties binnen de dienst Endocrinologie en het Centrum voor Seksuologie en Gender (CSG) wel mogelijke settings waarbinnen patiënten kunnen geworven worden, indien ze in de eerste fase van de TLS strategie worden geselecteerd. Tijdens vooraf bepaalde momenten waarop transgender patiënten worden gezien zal dan de behandelend arts of psycholoog van het CSG een vooraf bepaald aantal personen vragen of men wil deelnemen aan de studie (bijvoorbeeld: elke derde patiënt). Indien patiënten mondeling toestemming geven aan hun hulpverlener om deel te nemen aan de studie worden ze na de consultatie doorgestuurd naar een aparte ruimte waar de onderzoeker de studie in detail uitlegt en de stappen op het swab2know platform worden doorlopen, analoog aan de dataverzameling in niet-klinische fysieke settings.

1. Wanneer verwacht men voordeel voor de deelnemer?

a. Heeft het experiment een diagnostisch of therapeutisch doel dat onmiddellijk voordeel voor
de onderzochte zal brengen?

ja, kennis HIV status  neen

b. Maakt het experiment deel uit van een diagnostisch en therapeutisch plan waarvan men mag
verwachten dat de resultaten binnen afzienbare tijd voor andere zieken nuttig zullen zijn?

ja  neen

c. Maakt het experiment deel uit van een geheel van onderzoeken waarvan het diagnostische
of therapeutische belang niet onmiddellijk duidelijk is, maar er mag worden verwacht dat de
resultaten later tot diagnostische of therapeutische toepassingen of tot een betere kennis van
de fysiopathologische mechanismen zullen leiden?

ja  neen

1. Welke interventies zijn specifiek voor de studie (naast de standaardbehandelingen),
   hoe frequent en gedurende welke tijd?

a. Zuiver klinische evaluaties, om de

b. Functietests of dynamische proeven

Welke

Om de

c. Radiografische en/of isotopische investigaties

Welke

Om de

d. Bloedafnamen:

e. Weefselafname:

f. Andere: eenmalige speekseltest afgenomen door de participant zelf, gecombineerd met korte vragenlijst (5 min) + indien toestemming een opvolgvragenlijst (10 min) 2 weken na afname speekseltest

1. Rekening houdend met de huidige gegevens van de wetenschap

a. Meent u dat deze studie:

waarschijnlijk geen enkel risico inhoudt

een mogelijk risico inhoudt.

Welk risico en de frequentie:

zeer waarschijnlijk een risico inhoudt.

Welk risico en de frequentie:

b. Welke zijn de meest voorkomende bijwerkingen van het preparaat onder studie?

(de bijwerkingen moeten ook duidelijk vermeld worden in het informatie- en toestemmingsformulier van de deelnemer)

Niet van toepassing.

1. Informatie en toestemming van de proefpersonen

a. Wilsbekwame volwassenen

ja  neen

Wordt de toestemming van de proefpersonen verkregen na een klare en objectieve uiteenzetting van

het doel van het onderzoek?

Schriftelijk:

ja  neen

Mondeling:

ja  neen

Zo neen, waarom niet?

Wordt in dat laatste geval de toestemming gegeven door anderen dan de proefpersonen?

ja  neen

Zo ja, door wie?

Zijn er speciale groepen: eigen studenten, eigen personeel?

neen

b. Wilsonbekwame volwassenen (= sommige psychiatrische patiënten, personen in de onmogelijkheid hun wil te uiten, …)

ja  neen

Wordt de toestemming gegeven door anderen dan de proefpersonen?

ja  neen

Zo ja, door wie?

c. Kinderen

ja  neen

Wordt de toestemming gevraagd van hun wettelijke verantwoordelijken?

ja  neen

Is er een informatie- en toestemmingsformulier voor kinderen vanaf 12 jaar voorzien?

ja  neen

1. Is het informatieformulier voor de proefpersonen in de bijlage gevoegd

ja  neen

Zo neen, waarom niet?

1. Is het formulier voor schriftelijke toestemming in de bijlage gevoegd?

ja  neen

Zo neen, waarom niet?

1. Zullen de personen in de loop van deze studie voortdurend onder medisch toezicht staan?

ja  neen

a. Wie is de toezichthoudende arts?

NVT

b. Zal dat toezicht, zo nodig, verzekerd kunnen worden tijdens de uren die op de studie volgen?

ja  neen

c. Als de persoon naar huis terugkeert tijdens de uren die op het onderzoek volgen, zal in geval van nood
snel contact met een arts kunnen opgenomen worden?

ja  neen

d. Naam van die arts?

NVT

1. Is er voor het onderzoek een verzekering afgesloten conform de Belgische wet van 7/5/2004?
   (het verzekeringscertificaat moet bij de aanvraag gevoegd worden indien niet verzekerd door UZ Gent/UGent)

ja

Door welke verzekeringspolis bent u verzekerd? (verwijzen naar een bijgevoegd document volstaat niet)

UZ Gent (UZ Gent-no fault)

UGent

andere + omvang van de dekking:

neen, waarom niet?

1. Einddatum experiment

Datum: zaterdag 31 december 2022

**Let wel: elk experiment op mensen na de einddatum is niet meer gedekt door de verzekering zodat
 op dat ogenblik u in overtreding bent met de wettelijke beschikkingen.
 U kan het experiment wel verlenging mits een nieuwe aanvraag.**

1. Financiële overeenkomst
   Indien een definitieve financiële overeenkomst nog niet beschikbaar is, kan een budgetvoorstel dat tegen­getekend is door een vertegenwoordiger van de financierder + onderzoeker volstaan). Indien het bedrag van de definitieve financiële overeenkomst hoger is dan het ingediende budgetvoorstel, moet die definitieve financiële overeenkomst alsnog ter goedkeuring voorgelegd worden aan de Commissie voor Medische Ethiek.

niet van toepassing

aanwezig met volgende onderverdeling:

ereloon:

vergoeding voor technische prestaties:

**Ik verklaar de gehele verantwoordelijkheid van het hierboven vermeld project op mij te nemen en bevestig dat voor zover de huidige kennis het toelaat, de inlichtingen met de werkelijkheid overeenstemmen.**

| De onderzoeker  **datum**  **naam**  T’Sjoen Guy  **handtekening** |  | Het UZ-diensthoofd of de vakgroepvoorzitter  (voor akkoord)  **datum**  **naam**  **handtekening** |
| --- | --- | --- |
| **Het UZ-diensthoofd of de vakgroepvoorzitter van eventuele andere betrokken diensten (voor akkoord)** | | |
| **datum**  **naam**  **handtekening** |  | **datum**  **naam**  **handtekening** |

| **ANNEX – Verkrijgen/gebruik menselijk lichaamsmateriaal (MLM)** |
| --- |
|  |

Dit formulier is bedoeld voor onderzoekers die stalen van menselijk lichaamsmateriaal (MLM) collecteren, bewaren en/of gebruiken tijdens de studie. Gelieve deze annex ook in te vullen wanneer de stalen tijdens de studie onmiddellijk geanalyseerd worden en niet worden opgeslagen. **Opgelet, voor de aanmelding van een nieuwe prospectieve biobank dient een Document F te worden ingevuld.**

**Wat is voor deze studie van toepassing?**

**Indien u MLM zal verzamelen bij deze studie en u ook reeds verzameld materiaal zal gebruiken, gelieve zowel optie 1 als 2 te selecteren.**

**Indien u enkel MLM wenst te gebruiken dat verzameld wordt via een prospectieve biobank met “partially broad” ICF of via een andere FAGG erkende biobank (geen collectie bij deze studie), gelieve enkel optie 2 aan te duiden.**

1) Nieuwe collectie, nog te verzamelen menselijk lichaamsmateriaal.

Er zal gebruik gemaakt worden van:

Studie-specifieke U(Z)Gent biobank, nieuwe aanmelding. **Vul DEEL A, B en D in.**

Reeds aangemelde prospectieve U(Z)Gent biobank. **Vul DEEL B en D in.**

Naam biobank: Labo Endocrinologie 6K12

Medisch beheerder: Prof. Dr. Guy T’Sjoen

Referentie Commissie voor Medische Ethiek: BR-96

FAGG aanmeldingsnummer: BB190131

Andere FAGG erkende biobank. **Vul DEEL B en D in.**

Naam biobank: ITG Biobank

Medisch beheerder: dr. M. van Frankenhijsen

Referentie Commissie voor Medische Ethiek:

FAGG aanmeldingsnummer: BB190041

2) Gebruik van stalen uit reeds bestaande biobank(en). **Vul DEEL C en D in.**

3) Dit is een geneesmiddelenonderzoek (proef). **Vul enkel DEEL B en D in.**

Dit wil zeggen dat er geen bijkomende vereisten zijn wanneer materiaal wordt verzameld en gebruikt zoals beschreven in een aanvraag om klinische proef goedgekeurd door het FAGG en een ethisch comité. Wanneer de menselijke stalen verzameld in het kader van klinische proeven worden aangewend voor een ander doel dan voorzien in het goedgekeurde dossier moeten deze evenwel worden overgemaakt aan een biobank.

DEEL A: Aanmelding studie-specifieke biobank

DEEL B: Nieuwe collectie, nog te verzamelen menselijk lichaamsmateriaal

DEEL C: Gebruik van stalen uit reeds bestaande biobank(en)

DEEL D: Algemeen

________________________________________________________________________________

DEEL A: Aanmelding studie-specifieke biobank

NVT

____________________________________________________________________________

DEEL B: Nieuwe collectie, nog te verzamelen menselijk lichaamsmateriaal

1. **Nieuw te collecteren MLM**

Amnion vocht

Bloed, **max volume(ml)/deelnemer:**

Serum

Plasma

Navelstrengbloed

Beenmerg aspiraten, **max volume(ml)/deelnemer:**

Dried bloodspots

Stoelgang

Urine

Speeksel, sputum, mondswabs

Moedermelk

Cerebrospinaal vocht/pleuraal vocht/synoviaal vocht, **max volume(ml)/deelnemer:**

Follikelvocht

Traanvocht

Zweet

Kweekdruppels of cultuurmedia aspiraten

Cellen

zaadcellen

eicellen

RBC

PBMCs

bloedplaatjes

buccale cellen

Andere: :

Cellijnen

primaire cellijnen

commerciële cellijnen

Haar, lichaamhaar, haarfollikel

DNA (genomisch DNA, cDNA, cell-free DNA

RNA (miRNA, siRNA)

Eiwitten

Reproductief materiaal (gonaden, embryo's, foetussen): hoeveel/deelnemer:

Weefsel

fresh frozen

FFPE

Andere:

1. **Wat is de traceerbaarheid van het materiaal?**

De stalen zijn direct identificeerbaar (rijksregisternummer, adremanummer, naam, geboortedatum, initialen,…)

De stalen worden gecodeerd/gepseudonimiseerd (code zonder identificerende gegevens, te herleiden tot de deelnemer indien men over de link beschikt)

De stalen worden geanonimiseerd (link met deelnemer wordt volledig doorbroken)

Geeft de deelnemer toestemming voor anonimisering in het ICF (dit is verplicht)?

Ja  Neen

________________________________________________________________________________

DEEL C: Gebruik van stalen uit reeds bestaande biobank(en)

NVT

________________________________________________________________________________

DEEL D: Algemeen

1. **Gelieve een overzicht van de flow van het MLM bij deze studie te geven. Waar gebeurt de afname, waar gaan de stalen heen, waar gebeurt welke analyse, waar worden de stalen bewaard/vernietigd…? Elke stap moet duidelijk beschreven worden. Gebruik eventueel ook figuren. Geef duidelijk aan wat behoort tot de ‘standard of care’ en wat studie-specifiek is.**

Speekselstalen worden op twee manieren verzameld en telkens gekoppeld aan een vragenlijst in te vullen op de website [www.swab2know.eu](http://www.swab2know.eu) waar deelnemers toestemming geven :

1. Via self-sampling@home. Na registratie krijgen deelnemers een speekseltest opgestuurd en verzenden die naar het ITG in een voorgefrankeerde enveloppe. De stalen geworven via de online sampling methode komen rechtstreeks in de ITG biobank terecht.
2. Dataverzameling binnen een fysieke setting. Ook hier nemen deelnemers zelf een speekselstaal af en vullen via een tablet de vragenlijst op de website [www.swab2know.eu](http://www.swab2know.eu) in.Stalen worden verzameld door de onderzoekers van UZGent en tijdelijk opgeslagen in respectievelijk de biobank van UZGent en worden daarna verzonden naar de ITG biobank.

Alle stalen worden uiteindelijk in de ITG biobank verzameld waar ze 5 jaar bewaard zullen worden.

Volgende testen worden op de stalen uitgevoerd: HIV antilichamen worden opgespoord aan de hand van DPP HIV1/2 Assay (Chembio Diagnostic Systems, Inc).

In geval de speekseltest reactief is zal de uitslag via een bloedtest geconfirmeerd worden. Deelnemers worden doorverwezen naar hun huisarts of arts naar keuze. **Dit maakt deel uit van de standard routine care en is geen onderdeel van de studie.** ITG neemt hierbij een faciliterende rol op waarbij sommige stalen wel en andere niet op het ITG geconfirmeerd zullen worden. Deze confirmatie is onderdeel van de “standard of care”. Resultaten van de test worden opgevraagd maar niet geregistreerd als onderdeel van de studie. Stalen worden evenmin opgeslagen in een biobank.

1. **Gebruik van de stalen binnen de studie:**

Wordt er DNA/RNA geëxtraheerd uit de verkregen stalen?  ja   nee

Zo ja, wordt daarvoor geïnformeerde toestemming gevraagd aan de patiënt?  ja   nee

1. **Worden de stalen gebruikt in/getransfereerd naar:**

het binnenland (indien stalen verzameld door UZGent)

Specifieer de centra/labs/externen: transfer van biobank UZ Gent naar biobank ITG

het buitenland

Specifieer de centra/labs/externen:

NVT, geen transfer

1. **Beëindiging van de studie:**

**Gelieve te kopiëren indien voor verschillende types stalen iets anders wordt gedaan.**

Aan het einde van de studie worden de stalen (type stalen):

vernietigd 5 jaar na start van de studie.

overgedragen naar een andere aangemelde prospectieve biobank.

Gegevens erkende biobank:

Naam biobank:

Medisch beheerder:

Referentie Commissie voor Medische Ethiek:

FAGG aanmeldingsnummer:

verder bewaard in de reeds aangemelde prospectieve biobank.

1. **Handtekening Medisch Beheerder**

**Voor de Biobank ITG**

Ik verklaar akkoord te gaan met deze studie, inclusief deze annex.

Gelezen, goedgekeurd en ondertekend

Naam: Maartje van Frankenhuijsen Datum:

Handtekening:

**Voor de Biobank UZ Gent Labo Endocrinologie 6K12**

Ik verklaar akkoord te gaan met deze studie, inclusief deze annex.

Gelezen, goedgekeurd en ondertekend

Naam: prof. Dr. Guy T’Sjoen Datum:

Handtekening:
