# Supplementary material for: Prevalence and associated risk factors of HIV infections in a representative transgender and non-binary population in Flanders and Brussels (Belgium): Protocol for a community-based, cross-sectional study using time-location sampling
Source: PLoS One. 2022 Apr 11;17(4):e0266078. doi: 10.1371/journal.pone.0266078 (PMC9000107; doi:10.1371/journal.pone.0266078)
Supplement: S4 File — (DOCX) [file pone.0266078.s007.docx]

| Document AINTERVENTIONAL ACADEMIC RESEARCH | | | |
| --- | --- | --- | --- |
|  | | | |
| **MEDICAL ETHICS COMMITTEE** | | | |
| **phone**  +32 (0)9 332 56 13 \| +32 (0)9 332 33 36 \| +32 (0)9 332 68 55 | | **fax**  +32 (0)9 332 49 62 | **e-mail**  ethisch.comite@uzgent.be |
|  |  | | |

**REQUEST FOR ADVICE FROM THE MEDICAL ETHICS COMMITTEE ON HUMAN RESEARCH**



**PROJECT EUDRACT NUMBER** (if interventional drug research ): N/A

1. **Title of the study**

**Prevalence and associated risk factors of HIV infections in a representative transgender and non-binary population in Flanders (Belgium): a community-based, cross-sectional study using time-location sampling**

1. **Data of the researcher(s)
   [the first researcher must be a person who is permanently attached to the service (not ASO) or university]**

Name: T'Sjoen First name: Guy

Position: Full professor and head

Center: Department of Endocrinology: Center for Sexology and Gender

Faculty/department : Faculty of Medicine and Health Sciences - Department of Internal Medicine and Pediatrics

Telephone/mobile phone: 093322107

E-mail: guy.tsjoen@uzgent.be

Name head of department or department chair: Prof. Dr. Guy T'Sjoen

Name: Motmans First name: Joz

Position: Scientific collaborator

Center: Department of Endocrinology: Center for Sexology and Gender: Transgender Infopunt

or faculty/department:

Telephone/mobile phone: 093321178

E-mail: joz.motmans@uzgent.be

Name head of department or department chair: Prof. Dr. Guy T'Sjoen

Details of the employee(s) involved in the study

Name: Van Schuylenbergh First name: Judith

Position: Scientific collaborator

Center: Department of Endocrinology: Center for Sexology and Gender: Transgender infopunt

Telephone/mobile phone: 093325725

E-mail: judith. vanschuylenbergh@uzgent.be

Name UZ department head or department: Prof. Dr. Guy T'Sjoen

1. **Type of research**

☒ interventional research

☐ with drugs (indicate all items that apply)

☐ phase I

☐ phase II

☐ phase III

☐ phase IV

☐ trial for gene therapy and somatic cell therapy

☐ trial with medicines containing genetically modified organisms

☐ trial with cell therapy with xenogens

☒ other

☐ medical device

☐ blood collection, RX, …

☒ other: saliva collection and questionnaires

1. **Is the research**

☐ diagnostic ☐ therapeutic

☐ physiological ☐ physiopathological

☐ morphological ☒ epidemiological

1. **Is the research in Belgium**

☒ monocentric

☐ multicentric

☐ the UZ Gent Medical Ethics Committee is the central committee

☐ yes (name, address, tel, fax and e-mail of other Medical Ethics Committees

participating in the study + name of the local investigator)

☐ no (name, address, tel, fax and e-mail of the Central Committee on Medical Ethics)

1. **Will this study also take place abroad N/A**

☐ in Europe – Which are the participating countries:

☐ in the United States

1. **Is this research project financially supported?**

☒ yes ☐ no

☐ FWO/BOF

☐ pharmaceutical industry:

☒ other: Belgian Red Cross Flanders, Institute for the Equality of Women and Men (IGVM), Gilead (see appendices 6, 7 and 8)

1. **Who** **is the sponsor of the study who is not is industry sponsored?**

☒ employee of UZ Gent (name and address): T'Sjoen Guy

☐ employee of UGent (name and address):

☐ other, specify (name and address):

1. **Provide a short summary of the protocol (minimum 30 sentences/one half page and maximum one page),** **intelligible to people not specialized in the matter. Do not just refer to an attached protocol.**

International research shows that transgender people are at high risk for infection with HIV. However, European and Flemish research is completely lacking. It is of note that regional policy, such as the blood donation policy of Belgian Red Cross-Flanders (co-funder of this study), which currently excludes transgender people from blood donation, is based on non-Belgian and even non-European HIV prevalence rates. Since there is no scientific basis for such an exclusion policy in Flanders, this can be regarded as discrimination based on gender identity. In this study we want to map the HIV prevalence in Flemish transgender and non-binary people, and associated risk factors.

Because the transgender community in Flanders is incoherent and scattered, a **preparatory study** was first set up to map transgender communities (see EC application BC-08157). This study forms the basis for the sampling frame of the HIV prevalence study and ensures that a more representative sample can be obtained. The HIV prevalence study uses a 2-stage Time Location Sampling (TLS) frame: a number of settings will randomly be selected from the sampling frame; and, within each selected setting, a certain number of respondents are randomly selected to participate in the study. Settings can be categorized as physical settings (for example: events, cafes, support group meetings, practice of care providers) or online settings (for example: Facebook groups, Discord forums).

Based on **saliva** tests (and if reactive confirmed with a blood test if possible) the HIV prevalence within the transgender population will be estimated. The study is being conducted in collaboration with the Institute of Tropical Medicine (ITG) and uses the online platform [www.swab2know.eu](http://www.swab2know.eu), which is already being used for a similar study on HIV in Men who have Sex with Men (MSM). After reading the information and providing consent (see appendix 3 and 4), participants must first register with their e-mail address (see below), after which they must complete a short questionnaire (see appendix 1) and asked to take a saliva sample themselves.

The study is carried out/performed by the Transgender Infopunt (UZ Gent) because this is a well-known and trusted information and expertise center for the target population. A study-specific page will be developed on the website [www.transgenderinfo.be](http://www.transgenderinfo.be), where additional context of the study can be found (see appendix 5). Participants are recruited in both physical and online settings.

Data collection within physical settings takes place via a tablet on which the participant goes through the questionnaire on the swab2know platform. The participant takes a saliva sample himself and provides it to the researcher. Within 5 days, the researcher sends the collected saliva samples to ITG, where the samples are processed and analyzed (see appendix 9). At large physical settings (parties, events) or within more difficult-to-reach parts of the target population (for example: people of color, sex workers), the researcher may be assisted for the data collection by a *peer recruiter* or key figure within the transgender community.

Online data collection takes place directly via the platform [www.swab2know.eu](http://www.swab2know.eu). Participants will receive a saliva test and must send it to ITG in a pre-paid envelope.

Inclusion criteria for participation are: adults identifying as transgender or non-binary (self-identification) and living in Flanders or Brussels.

The study has the advantage for participants that they **can request the result of the HIV test** a few weeks after participation. Therefore, they need to register with an email address. The ITG employee enters the results, after which the participants receive an email with the notification that they can consult their results via the swab2know platform. Participants with a reactive result will be referred to their GP or a doctor of their choice for a confirmation test via blood draw. ITG provides follow-up and linkage to care: referral is part of the standard routine care and is not part of the study, but ITG does play a facilitating role. See also: <https://www.swab2know.eu/BE/N/hoewerkthet/>

Two weeks after providing the saliva sample, participants will be asked by e-mail to participate in a follow-up questionnaire (see appendix 2), if they gave permission to be contacted.

All data will be pseudonymized and linked via a code system, to which only (i) the principal investigator and the (ii) ITG employee, who sends the envelopes, processes the test results and finalizes the communication to the participant, will have access. All other researchers will have only access to a pseudonymised database. The saliva samples are coded analyzed.

The data will be analyzed by the researcher affiliated with UZ Gent (Judith Van Schuylenbergh) and the researcher affiliated with the Center for Evidence-Based Practice (CEBaP) of the Belgian Red Cross-Flanders (Niels De Brier). The results of the study will be disseminated in a widely accessible report commissioned by the Institute for the Equality of Women and Men (IGVM) as well as in scientific articles.

An extensive draft protocol of this study can be found in appendix 10.

1. **What are the arguments (theoretical, experimental or other) that suggest an advantage of the new method, preparation, ... to be tested over the known and already used ones?**

International research suggested that very high HIV prevalence rates may exist among transgender people, but European research on this topic is completely lacking. Most of the existing research was conducted in the metropolitan context of San Francisco or New York, or Asian countries where the social context for transgender people is very different from the European context. Moreover, the current research is limited by a selective focus on specific subgroups of the transgender population (e.g. transgender women, sex workers) and convenience sample methods were commonly used providing samples with disproportionately many high-risk persons (sexual health clinics, community centers, prostitution neighborhoods). (See also Van Schuylenbergh, Motmans & Coene, 2018).

The World Health Organization (WHO) relies on this selective and unrepresentative research for underpinning their guidelines for HIV prevention and treatment in 5 key populations: MSM, drug injectors, inmates in closed institutions, sex workers and transgender persons (WHO, 2014). The Belgian Red Cross-Flanders also relies on this research to set up exclusion criteria for blood donation for transgender people. In more concrete terms, initially the same rules apply to blood donation for transgender people as for MSM, for which there is sufficient research to scientifically underbuilt a (partial) exclusion. However, given the lack of representative data that is valid for the Flemish transgender population, the exclusion of transgender people can be seen as discrimination.

The aim of this study is to estimate the HIV prevalence in a representative sample of Flemish transgender people. In the first instance, the results of the study will directly serve as a knowledge base to scientifically underpin the blood donation policy in Flanders. The Belgian Red Cross-Flanders is therefore an important partner in this study, together with the Institute for the Equality of Women and Men (IGVM). Moreover, it will also lead to a more nuanced HIV prevalence rate for the broad and diverse transgender population, and provide insights into different risk groups within this very diverse transgender population. The design for this study is unique within the current research on HIV in transgender people, because of the large sample size, the use of the TLS method and the use of laboratory-confirmed HIV data via saliva tests at this scale. We aim to put HIV research in transgender people on the European research agenda.

References:

World Health Organization (2014).[*Consolidated guidelines on HIV prevention, diagnosis, treatment and care for key populations.*](https://www.who.int/hiv/pub/guidelines/keypopulations/en/)Report, WHO.

Van Schuylenbergh, Motmans & Coene (2018).[Transgender and non-binary persons and sexual risk: A critical review of 10 years of research from a feminist intersectional perspective](https://journals.sagepub.com/doi/full/10.1177/0261018317732478).*Critical Social Policy,*38:2.

1. **Has an analogous study already been conducted elsewhere, in whole or in part?**

If so, where? What was the result? Why is it reintroduced in this study?

The Institute of Tropical Medicine (ITG, Antwerp), one of the partners in the research, has a lot of experience with the methods that will be used in the current research project. ITG has been using these methods for years for their research on HIV among Men who have Sex with Men (MSM) on the one hand and Sub-Saharan Migrants (SAM) on the other hand. These research projects concern target populations that are substantially different from the transgender population that will be investigated in the proposed study. This study is innovative within the existing HIV literature as well as the current state-of-the-art on the health of transgender people.

The Time-Location Sampling (TLS) strategy, the use of peer recruiters/key figures and the use of the saliva tests in combination with a questionnaire via a tablet was successfully applied in the 'Together project', a study on HIV in Sub-Saharan Migrants (SAM) in Antwerp (Nöstlinger & Loos, 2016; Loos et al., 2016; Loos et al., 2017).

The protocol for the data collection of the current study was based on the ITG study 'Swab2know'. Swab2know is an HIV prevention project targeting Men who have Sex with Men (MSM). This study uses the platform [www.swab2know.eu](http://www.swab2know.eu) on which people can request an HIV saliva test free of charge and anonymously. The researchers also go to clubs, saunas and parties at certain times in the evenings and on weekends to offer free and anonymous saliva tests. The method used was found to be effective and positively received within the original target population of MSM (Platteau et al., 2015). The study 'Swab2know' is mainly focused on HIV prevention (increasing testing behaviour) and, besides the different target population, this is the biggest difference with the current study, which aims to map the HIV prevalence rate within a hitherto poorly studied population in Flanders.

References:

Loos, J., Nöstlinger, C., Vuylsteke, B., Deblonde, J., Ndungu, M., Kint, I., . . . Laga, M. (2017). First HIV prevalence estimates of a representative sample of adult sub-Saharan African migrants in a European city. Results of a community-based, cross-sectional study in Antwerp, Belgium. *Plos one*, 12(4), e0174677.

Loos, J., Vuylsteke, B., Manirankunda, L., Deblonde, J., Kint, I., Namanya, F., . . . Adobea, D. (2016). Together project to increase understanding of the HIV epidemic among Sub-Saharan African migrants: Protocol of community-based participatory mixed-method studies. *JMIR research protocols*, 5(1), e48.

Nöstlinger, C., & Loos, J. (2016). Involving lay community researchers in epidemiological research: experiences from a seroprevalence study among sub-Saharan African migrants. *AIDS care,* 28(sup1), 119-123.

Platteau, T., Fransen, K., Apers, L., Kenyon, C., Albers, L., Vermoesen, T., ... & Florence, E. (2015). Swab2know: an HIV-testing strategy using oral fluid samples and online communication of test results for men who have sex with men in Belgium. *Journal of medical Internet research*,*17*(9), e213.

1. **Will a chemical substance be administered?**

☐ yes ☒ no

If yes:

a. In what way?

b. Name and origin of the substance:

c. Who is responsible for the reception, storage, distribution and return of unused chemical

substances?

d. Will radioisotopes be administered?

☐ yes ☒ no

Which one?

1. **If it concerns a new substance: has the researcher taken cognizance of the complete toxicological, animal pharmacological and human file? N/A**

☐ yes ☐ no

If no, please explain:

1. **Choice of subjects:**

a Healthy?

☒ yes ☐ no

Patients suffering from:

b. Pregnant women or women who could become pregnant during the study?

☐ yes ☒ no

c. Number of test subjects in UZ Gent: 800

d. Number of external test subjects (in Belgium): 600

Number of test subjects within Belgium = 800 + 600 = 1400

**Attention: the experiment is only insured for the number specified here.**

**If you want to include additional participants, you will have to request this via an amendment.**

e. Age: adults (18+)

f. Gender: m/f/x

g. How are they recruited?

Participants in non-clinical physical settings are addressed by the researcher, who reviews the inclusion criteria (18 years or older, identifying as transgender or non-binary and living in Flanders or Brussels) and explains the purpose of the study. After giving verbal consent, the participant is asked to go through the different steps on the swab2know platform on the tablet. First, participants are given all the information and the written informed consent form, after which they have to give digital permission again to continue with the questionnaire and instructions for taking the saliva test.

Afterwards, participants will receive a card with contact details of the research team, a link and QR code to the study-specific pages on the website [www.transgenderinfo.be](http://www.transgenderinfo.be), where all information about the study can be found and where updates will be posted about the study and the results. .

Participants who are recruited online are approached by the researcher or peer recruiter, who sends them the link of the swab2know platform, on which they can go through the steps themselves and receive the saliva test sent at home. After the study, they are also forwarded to the study-specific pages on the website [www.transgenderinfo.be](http://www.transgenderinfo.be).

Participants will have option to enter their contact details in the questionnaire in order to be kept informed about the progress and results of the study. If participants give permission for this communication, they will receive an update several times during the project via email (maximum 5 mailings).

**No patient data is used. Participants are not necessarily patients of UZ Gent.**

Within UZ Gent, the consultations within the Endocrinology department and the Center for Sexology and Gender (CSG) are possible settings in which patients can be recruited, if they are selected in the first phase of the TLS strategy. During predetermined moments when transgender patients are seen, the treating physician or psychologist of the CSG will then ask a predetermined number of people if they want to participate in the study (random order). If patients give verbal permission to their healthcare provider to participate in the study, they are sent to a separate room after the consultation where the researcher explains the study in detail and the steps on the swab2know platform are completed, analogous to the data collection in non-trial clinical physical settings.

1. **When are benefits for the participant expected?**

a. Does the experiment have a diagnostic or therapeutic purpose that will bring immediate benefit to
the subject?

☒ yes, knowledge of HIV status ☐ no

b. Is the experiment part of a diagnostic and therapeutic plan whose results can be expected to be useful to other patients in the foreseeable future?

☐ yes ☒ no

c. Is the experiment part of a set of studies whose diagnostic or therapeutic importance is not immediately apparent, but it can be expected that results will later to lead to diagnostic or therapeutic applications or to a better knowledge of the physiopathological mechanisms?

☒ yes ☐ no

1. **Which interventions are specific to the study (besides the standard treatments),
   how frequently and during what time?**

a. Purely clinical evaluations, every …

b. Functional tests or dynamic tests

Which

Every

c. Radiographic and/or Isotopic Investigations

Which

Every

d. Blood samples:

e. Tissue collection:

f. **Other: one saliva test administered by the participant himself, combined with a short questionnaire (5 min) + if consented, a follow-up questionnaire (10 min) 2 weeks after taking the saliva test**

1. **Taking into account the current data from science**

a. Do you think this study:

☒ probably not at all risky

☐ poses a potential risk.

What risk and frequency:

☐ very likely to be a risk.

What risk and frequency:

b. What are the most common side effects of the preparation under study?

(the side effects must also be clearly stated in the participant's information and consent form)

Not applicable.

1. **Information and consent of the subjects**

a. Competent adults

☒ yes ☐ no

Is the consent of the subjects obtained after a clear and objective statement of the purpose of the study?

Written:

☒ yes ☐ no

Oral:

☒ yes ☐ no

If no, why not?

In the latter case, is the consent given by others than the test subjects?

☐ yes ☐ no

If yes, by whom?

Are there special groups: own students, own staff?

no

b. Incapacitated adults (= some psychiatric patients, persons unable to express their will, ...)

☐ yes ☒ no

Is consent given by others than the test subjects?

☐ yes ☒ no

If yes, by whom?

c. Children

☐ yes ☒ no

Is permission requested from their legal guardians?

☐ yes ☒ no

Is there an information and consent form for children from 12 years old?

☐ yes ☒ no

1. **Is the information form for the subjects included in the appendix**

☒ yes ☐ no

If no, why not?

1. **Is the form for written consent attached?**

☒ yes ☐ no

If no, why not?

1. **Will the individuals be under continuous medical supervision during the course of this study?**

☐ yes ☒ no

a. Who is the supervising physician?

N/A

b. Can such supervision, if necessary, be ensured during the hours following the study?

☐ yes ☒ no

c. If the person returns home during the hours following the examination, can doctor be contacted quickly in case of emergency?

☐ yes ☒ no

d. Name of that doctor?

N/A

1. **Has insurance been taken out for the examination in accordance with the Belgian law of 7/5/2004?
   (the insurance certificate must be enclosed with the application if not insured by UZ Gent/UGent)**

☒ yes

Which insurance policy covers you? (referring to an attached document is not sufficient)

☒ UZ Gent (UZ Gent-no fault)

☐ UGent

☐ other + scope of cover:

☐ no, why not?

1. **End date of experiment**

date: Saturday, December 31, 2022

**Please note: any experiment on humans after the end date is no longer covered by the insurance, so that at that time you are in violation of the legal provisions. You can extend the experiment with a new application.**

1. **Financial agreement
   If a final financial agreement is not yet available, a budget proposal signed by a representative of the funder + researcher may suffice. If the amount of the final financial agreement is higher than the budget proposal submitted, that final financial agreement must still be submitted to the Medical Ethics Committee for approval.**

☒ not applicable

☐ present with the following subdivision:

☐ fees:

☐ compensation for technical performance:



**I declare that I take full responsibility for the project mentioned above and confirm that, to the best of current knowledge, the information corresponds to reality.**

| **The researcher**  **date**  ****  **name**  **T'Sjoen Guy**  **signature**  **** |  | **The UZ department head or department chair**  **(for approval)**  **date**  ****  **name**  ****  **signature**  **** |
| --- | --- | --- |
| **The UZ department head or department chair of any other services involved (for approval)** | | |
| **date**  ****  **name**  ****  **signature**  **** |  | **date**  ****  **name**  ****  **signature**   |

| **ANNEX – Obtain/use human body material (HBM)** |
| --- |
|  |

This form is intended for researchers who collect, store and/or use samples of human body material (HBM) during the study. Please also complete this annex if the samples are immediately analyzed during the study and not stored. **Please note that for the registration of a new prospective biobank a Document F must be completed.**

**What applies to this study?**

**If you will be collecting HBM in this study and will also use material already collected, please select both option 1 and 2.**

**If you only wish to use HBM that is collected via a prospective biobank with “partially broad” ICF or via another FAMHP recognized biobank (no collection in this study), please only indicate option 2.**

☒ 1) New collection, human body material yet to be collected.

Use will be made of:

☐ Study-specific U(Z)Ghent biobank, new registration. **Complete PART A, B and D.**

☒ Already registered prospective U(Z)Ghent biobank. **Complete PART B and D.**

Biobank name: Lab Endocrinology 6K12

Medical administrator: Prof. dr. dr. Guy T'Sjoen

Reference Committee for Medical Ethics: BR-96

FAGG application number: BB190131

☒ Other FAMHP recognized biobank. **Complete PART B and D.**

Name of biobank: ITG Biobank

Medical manager: Dr. M. van Frankenhijsen

Reference Committee for Medical Ethics:

FAGG application number: BB190041

☐ 2) Use of samples from existing biobank(s). **Complete PART C and D.**

☐ 3) This is a drug trial (trial). **Only fill in PART B and D.**

This means that there are no additional requirements when material is collected and used as described in a clinical trial application approved by the FAMHP and an ethics committee. However, when the human samples collected in the context of clinical trials are used for a purpose other than that foreseen in the approved dossier, they must be transferred to a biobank.

PART A: Registration of a study-specific biobank

PART B: New collection, human biological material to be collected

PART C: Use of samples from existing biobank(s)

PART D: General information

________________________________________________________________________________

PART A: Registration of a study-specific biobank biological material

N/A

____________________________________________________________________________

PART B: New collection, human body material to be collected

1. **New HBM to be collected**

☒ Saliva, sputum, oral swabs

1. **What is the traceability of the material?**

☐ The samples are directly identifiable (national registry number, address number, name, date of birth, initials,…)

☒ The samples are coded/pseudonymized (code without identifying data, traceable to the participant if the link is available)

☐ The samples are anonymized (link with participant is completely broken)

Does the participant give permission for anonymization in the ICF (this is mandatory)?

☐ Yes ☐ No

________________________________________________________________________________

PART C: Use of samples from existing biobank(s)

N/A

________________________________________________________________________________

PART D: General information

1. **Please provide an overview of the flow of the HBM in this study. Where does the collection take place, where do the samples go, where does which analysis take place, where are the samples stored/destroyed…? Each step must be clearly described. Use figures if necessary. Indicate clearly what belongs to the 'standard of care' and what is study-specific**

Saliva samples are collected in two ways and each time linked to a questionnaire to be completed on the website www.swab2know.eu where participants give permission:

1. Via self-sampling@home. After registration, participants receive a saliva test and send it to the ITG in a prepaid envelope. The samples collected via the online sampling method end up directly in the ITG biobank.
2. Data collection within a physical setting. Here too, participants take a saliva sample themselves and fill in the questionnaire on the website www.swab2know.eu via a tablet. Samples are collected by the researchers at UZGent and temporarily stored in the UZGent biobank, after which they are sent to the ITG biobank.

The following tests are performed on the samples: HIV antibodies are detected using the DPP HIV1/2 Assay (Chembio Diagnostic Systems, Inc).

In case the saliva test is reactive, the result will be confirmed by a blood test. Participants are referred to their primary care physician or physician of choice. This is part of standard routine care and is not part of the study. ITG plays a facilitating role in this process, whereby some samples will and others will not be confirmed at ITG. This confirmation is part of the “standard of care”. Results of the test are requested but not recorded as part of the study. Samples are also not stored in a biobank.

1. **Use of samples in the study:**

Will DNA/RNA be extracted from the collected samples? ☐ yes ☒no

if so, is informed consent requested from the patient? ☐ yes ☐ no

1. **Will the sample be used in or transferred to:**

☒ Belgium (when samples are collected bij UZ Gent)

Specify the centers/labs/externals: transfer from biobank UZ Gent to biobank ITG

☐ Abroad

Specify the centers/labs/externals:

☐ N/A, no transfer

1. **Completion of the study:**

After the end of the study, the samples will be:

☒ destroyed 5 years after the start of the study.

☐ transferred to another registered prospective biobank.

Records of recognized biobank:

Biobank name:

Medical administrator:

Reference Committee for Medical Ethics:

FAGG application number:

☐ further stored in the same registered prospective biobank.

1. **Signature medical administrator**

**For Biobank ITG**

☐ I declare that I agree with this study, including this annex.

Read, approved and signed

Name: Maartje van Frankenhuijsen Date:

Signature:

**For Biobank UZ Gent Lab Endocrinology 6K12**

☐ I declare that I agree with this study, including this annex.

Read, approved and signed

Nama: prof. Dr. Guy T'Sjoen Date:

Signature:
